# Modeling drug retention as memory effects in obese patients using fractional and augmented models

**DOI:** 10.1038/s41598-025-10172-1

**Published:** 2025-07-19

**Authors:** Amani R. Ynineb, Erhan Yumuk, Dana Copot, Bora Ayvaz, Bouchra Khoumeri, Ghada Ben Othman, Marcian D. Mihai, Isabela R. Birs, Cristina I. Muresan, Clara M. Ionescu

**Affiliations:** 1 Department of Electromechanics, Systems and Metal Engineering, Research Group on Dynamical Systems and Control, Ghent University, Gent, 9052 Belgium; 2 Department of Control and Automation Engineering, Istanbul Technical University, Istanbul, 34469 Turkey; 3 Department of Automation, Technical University of Cluj-Napoca, Cluj-Napoca, 400027 Romania

**Keywords:** Fractional calculus, Pharmacokinetics, Compartmental models, Memory effect, Caputo derivative, Biomedical engineering, Applied mathematics

## Abstract

Lipophilic anesthetic drugs accumulate in adipose tissue, leading to delayed release and prolonged effects, particularly in obese patients. This study proposes two novel physiologically motivated pharmacokinetic (PK) models to address these dynamics. The first is an augmented model with a trap compartment to simulate retention, and the second is a fractional-order model using Partial-Caputo derivatives to capture memory effects. By applying a discrete-time Euler method to the augmented model, we reveal an inherent fading memory behavior, where the current drug release depends on a weighted influence of past drug concentrations in fat. Both models are integrated into a PK/PD framework. Their behavior is first explored in a single-input single-output (SISO) case using simulated Bispectral Index (BIS) responses under three common dosing protocols: single bolus, repeated boluses, and continuous infusion. Evaluation against real clinical data is then performed in a multiple-input single-output (MISO) case, where the simulated BIS responses are compared to recorded BIS measurements from a representative obese patient under total intravenous anesthesia (TIVA). During the awakening phase, both the augmented and fractional-order models reduce BIS prediction error compared to the classical model. The augmented model lowers RMSE by 22.5% (from 10.38 to 8.04), while the fractional model achieves a 21.4% reduction (to 8.16) (based on one obese patient case). Sensitivity analysis confirms the impact of the fractional-order parameter ($\alpha _{31}$) on long-term BIS dynamics. These results and proposed models illustrate the potential role of memory-aware PK models for advanced patient-specific digital twin systems in healthcare.

## Introduction

Accurate modeling of anesthetic drug dynamics is critical for maintaining optimal sedation levels and avoiding adverse effects during and after surgery. In clinical practice, most hypnotic and analgesic agents used in anesthesia are highly lipophilic^1^. These drugs initially circulate in the bloodstream and rapidly distribute to highly perfused tissues such as the brain, liver, and kidneys, where they exert their primary effects. They are then redistributed to less-perfused tissues such as muscle and fat. Once in the adipose tissue, however, lipophilic drugs tend to accumulate because of their high solubility in lipids. This creates a reservoir effect, where the drug is gradually released back into the bloodstream over time. This delayed clearance contributes to prolonged drug activity and a slower “washout” period, particularly in obese patients^2,3^.

The reservoir effect in adipose tissue introduces complexity in pharmacokinetic (PK) behavior, particularly in obese patients. This phenomenon reflects a form of history-dependent dynamics, where the system’s future states are influenced not only by its current state but also by its past. In this context, adipose tissue retains a “memory” of prior drug exposures, such that previously accumulated drugs continue to affect future release into the bloodstream^4^. This delayed and sustained release introduces challenges in accurately predicting drug concentrations over time, making anesthetic management during surgery more complex. It can also lead to prolonged sedation or other postoperative side effects^5^.

PK compartmental models are widely used to understand and predict how anesthetic drugs move through the body over time. These models simplify the body into compartments where the drug is absorbed, distributed, and eliminated. A key assumption underlying this approach is that each compartment is homogeneous, which means that drug concentration is considered uniform throughout each compartment^6^. This assumption, however, does not align with the current understanding of adipose tissue’s heterogeneity. As a result, classical compartmental models often overlook the complex dynamics of lipophilic drug distribution in fat, especially the delayed release and memory effects associated with adipose compartments.^7^.

Classical strategies to capture delay and memory in pharmacokinetics/pharmacodynamic (PK/PD) modeling include effect-site compartments, indirect response models, and delay differential equations (DDEs). Effect-site models introduce a hypothetical compartment to account for distribution delays between plasma and the site of action, governed by a first-order transfer rate. While effective for fast-acting drugs, they assume a single exponential timescale and often fail to capture long-term accumulation in peripheral tissues^8^. Indirect response and tolerance models incorporate intermediate biological processes (e.g., mediator turnover, receptor adaptation) to reflect pharmacodynamic memory, but they increase model complexity and are often mechanism-specific. DDEs impose fixed lags but cannot represent distributed or history-dependent delays, and pose identifiability and numerical challenges^9^. An alternative explanation for long-lived drug effects involves recycling between accessible and deep tissue compartments. The methadone study by Linares *et al.*^10^ provides a clear quantitative demonstration of this mechanism, showing how repeated uptake and release from peripheral tissues can sustain systemic drug levels over time.

In the literature, memory effects are often modeled using fractional calculus, which introduces non-local, history-dependent behavior into differential equations. Fractional calculus is highly versatile and has been applied in both engineering and biomedical fields. It has shown strong performance in areas such as advanced robotic control and pharmacokinetic modeling^11–13^. The typical approach is to modify standard ordinary differential equations by replacing integer-order derivatives with fractional-order operators. In compartmental models, this enables the incorporation of memory effects within each compartment^14,15^. However, to preserve mass balance, it is commonly assumed that all compartments have the same fractional-order^16–18^. Although this assumption ensures mathematical consistency, it does not reflect physiological reality, as blood and fat, for example, exhibit different degrees of heterogeneity.

The motivation of this work is to address the complex pharmacokinetics of anesthetic drugs in adipose tissue, particularly the memory effects associated with drug retention and delayed release. To overcome the limitations of the previously presented classical models, we propose two physiologically motivated extensions: (i) an augmented model with an additional trap compartment to represent drug accumulation and delayed release, and (ii) a fractional-order model that replaces integer derivatives with Partial-Caputo derivatives to incorporate memory effects. The augmented model preserves mechanistic interpretability, while the fractional model captures distributed memory across multiple timescales. Together, they form a flexible framework that bridges classical compartmental and memory-based modeling, particularly suited for describing the slow redistribution of drugs in obese patients. The Caputo formulation is particularly suitable for this application. It allows memory to be applied selectively to specific transitions (such as the slow release from fat into the bloodstream) while retaining classical dynamics elsewhere. Other formulations, such as Riemann–Liouville, Grünwald–Letnikov, or Caputo–Fabrizio, either require non-physical initial conditions, impose uniform memory across all compartments, or decay too quickly to represent long-term trapping effects. In contrast, Caputo derivatives are compatible with standard initial conditions and can capture heterogeneous retention with physiologically meaningful parameters.

Building on our previous work^19,20^, we further show that the Euler discretization of the augmented model reveals a fading memory kernel, closely resembling fractional dynamics. These two complementary modeling approaches enable a better representation of drug behavior in obese patients, where adipose tissue plays a dominant role in pharmacokinetic variability. In this context, the term *memory effect* refers to the influence of past drug concentrations on current system dynamics. This indicates that the system retains a history of previous states. *Fading memory* describes a specific type of memory effect where the influence of earlier concentrations decreases gradually over time.

The novelty of this study lies in the full development and comparative evaluation of two physiologically motivated modeling strategies to describe memory effects in anesthetic pharmacokinetics: an augmented model with a trap compartment, and a fractional-order model based on Partial-Caputo derivatives exclusively to describe the slow release of drug from the adipose. While our earlier work^21,22^ introduced the trap model and outlined the potential of fractional dynamics, this study implements both approaches in parallel. They are also integrated into a unified PK/PD framework and evaluated using clinical data. By systematically comparing their behavior and predictive performance, this study clarifies the complementary nature of both approaches and offers new insights into how drug accumulation in fat can be modeled mechanistically or phenomenologically. These insights suggest how future personalised dosing tools might be developed, as they account for inter-individual differences in adipose drug retention that are often overlooked in population-based models. In addition, this work contributes to the literature as a foundational step toward digital twins (DTs) in anesthesia by providing advanced PK/PD models that capture long-term drug dynamics and physiological variability.

To evaluate the proposed models, simulations are conducted using clinical data from a total intravenous anesthesia (TIVA) protocol. The models are tested in both single-input single-output (SISO) and multiple-input single-output (MISO) configurations, where simulated Bispectral Index (BIS) responses are evaluated against recorded BIS measurements. In the SISO case, three clinically relevant dosing strategies (single bolus, repeated boluses, and continuous infusion) are simulated to assess model behavior under different protocols. In the MISO case, model predictions are evaluated against real BIS data using quantitative performance metrics. Additionally, a sensitivity analysis is performed using the Morris method to investigate the influence of the fractional-order parameter ($\alpha _{31}$) on model output. Both the augmented and fractional-order models are compared to a classical three-compartment model, with particular attention to the induction and awakening phases. This comparative study highlights the physiological relevance of incorporating memory effects and adipose tissue heterogeneity into PK modeling.

The paper is organized as follows: Section 2 provides the physiological and clinical context, focusing on the challenges of drug accumulation and redistribution in adipose tissue during anesthesia. Section 6 introduces the modeling framework, including the classical three-compartment model, an augmented model with a trap compartment, and a fractional-order formulation based on Caputo derivatives. A pharmacodynamic component is also incorporated to link drug concentration to the hypnotic effect. Section 4 presents simulation-based validation using clinical BIS data, evaluating model performance across both single- and multi-drug scenarios during induction and awakening phases. Section 5 discusses the physiological and clinical implications of memory effects, particularly their relevance for DTs and closed-loop control. Section 6 outlines the methodological and modeling limitations of the current study. Section 7 proposes future extensions, including multi-drug interaction modeling and integration of dynamic physiological feedback. Finally, Section 8 concludes the paper by summarizing the key findings and their significance for anesthesia modeling.

## Context

### Accumulation of anesthetics and opioids in adipose tissue

Intravenous anesthetics and opioids often exhibit high lipophilicity, which leads to extensive distribution into adipose (fat) tissue. During prolonged infusions or repeated dosing, these drugs can accumulate in fat, creating a reservoir that slowly releases the drug back into circulation. This phenomenon contributes to prolonged drug action, a key aspect of pharmacokinetic “memory” where the body’s tissues retain drug over time. Clinically, such accumulation can lengthen the context-sensitive half-life (the time for drug concentration to decrease by 50% after stopping an infusion) and delay patient awakening from anesthesia or sedation.

A drug’s tendency to accumulate in fat is largely driven by its lipophilicity and resultant volume of distribution (Vd). Highly lipophilic agents leave the plasma and enter fatty tissues readily, yielding very large apparent Vd values^23^. When a lipophilic drug is given as a bolus, its clinical effect wanes as the drug redistributes from the well-perfused central compartment (blood and brain) into peripheral compartments (muscle and fat). With a continuous infusion, however, these peripheral compartments gradually fill. The longer the infusion, the more drug accumulates in fat. Once the infusion stops, drug in fat redistributes back into blood, prolonging the elimination phase. In practical terms, a drug that might have a 30-min half-time after a brief infusion can exhibit a half-time of several hours after a day-long infusion, due to the slow release from adipose stores^24^. Obesity amplifies these issues. Obese patients have a much higher fat mass (with relatively low blood perfusion), providing an even larger depot for lipophilic drugs. For modeling drug accumulation and redistribution in obese individuals, it is essential to distinguish between agents that significantly accumulate in adipose tissue and those that do not.

Several pharmacokinetic and clinical studies have documented the accumulation of lipophilic anesthetic and opioid agents in adipose tissue during prolonged sedation^23–25^. For example, modeling data have shown that the context-sensitive half-time of Propofol can extend from under 30 min to over 1–3 days after prolonged ICU infusions, due to redistribution from fat stores^24^. Similarly, Fentanyl exhibits sharply rising context-sensitive half-times with infusion duration, exceeding 6 h after continuous administration. These findings are supported by clinical studies showing higher drug concentrations in obese patients, attributed to enhanced fat storage^25^. An additional mechanism contributing to prolonged opioid retention is tissue-level recycling. Linares *et al.*^10^ demonstrated that methadone can accumulate in deep tissues, forming a large extravascular reservoir that slowly releases the drug back into circulation over multiple cycles. In contrast, drugs like Remimazolam and Remifentanil display minimal accumulation and remain context-insensitive even after extended infusions, due to rapid metabolism by esterases^26,27^. These studies underscore the need to account for adipose drug reservoirs in dosing decisions, especially during long surgeries or ICU sedation. Table 1 examine the commonly used drugs pharmacokinetic profile in Europe and their fat accumulation tendency. The focus was on the intravenous drugs. In contrast, inhalational Agents like Isoflurane and Sevoflurane both dissolve into fat over time, with fat-blood partition coefficients around 50^28^. However, due to low perfusion of adipose tissue, even in obese patients, the clinical impact of fat accumulation is limited for most surgery durations. Isoflurane, being more blood- and tissue-soluble, accumulates more and washes out slower than Sevoflurane. Sevoflurane offers faster awakening and is generally preferred for obese patients when inhaled agents are used.

**Table 1:** Summary of anesthetic and opioid drugs with respect to adipose tissue accumulation.

Drug	Class	Lipophilicity / Vd	logP	Context-Sensitive Half-Time	Traps in Fat
Propofol^24,25,29^	IV Anesthetic	High lipophilicity; Vd $\sim$300 L	3.8	Moderate increase (<30 min if <8 h)	✓
Ketamine^29,30^	IV Anesthetic	Lipophilic; Vd 2.5–5 L/kg	–	<40 min even after long infusion	✓
Midazolam^29,31^	Benzodiazepine	High lipophilicity; Vd increases in obesity	3.53	Prolonged with long infusion	✓
Fentanyl^32^	Opioid	Highly lipophilic; Vd $\sim$4 L/kg	4.05	Steep increase with infusion duration	✓
Sufentanil^32,33^	Opioid	Highly lipophilic; smaller Vd than Fentanyl	3.95	Shorter than Fentanyl; increases with duration	✓
Remimazolam^26^	Benzodiazepine	Moderate Vd (35–40 L); high clearance	–	<10 min; context-insensitive	✗
Etomidate^29,34^	IV Anesthetic	Lipophilic; large Vd	2.67	Minimal increase with duration	✗
Remifentanil^27^	Opioid	Small Vd; rapid esterase metabolism	1.25–1.75	$\sim$3–4 min; context-insensitive	✗
Morphine^32^	Opioid	Hydrophilic; Vd $\sim$3–4 L/kg	0.88	Moderate increase; metabolite-dependent	✗

### Overwiew on fractional-order in pharmacokinetic modeling

**Table 2:** Recent fractional-order models in pharmacokinetics (2022–2025).

#	Authors	Year	Fractional model used	Solving method	Application
1	Borkor et al.^35^	2025	Commensurable, non-commensurable, and implicit non-commensurable models	Fractional finite difference method (FFDM)	Amiodarone drug diffusion
2	Ramasamy et al.^36^	2025	p-Laplacian boundary value problems with modified Mittag-Leffler kernel	Hybrid numerical approach	Nonlinear and mixed pharmacokinetic models
3	Abdul-Aziz et al.^37^	2025	Population pharmacokinetic model	Monolix, Monte Carlo simulations	Caspofungin in critically ill patients on ECMO
4	Drapaca^38^	2024	Viscoelastic fractional model	Mathematical modeling	Donepezil hydrochloride neuronal effects
5	Xu et al.^39^	2024	fractional-order compartment models	Exact stochastic simulation method	General compartmental systems
6	Miskovic-Stankovic et al.^40^	2024	Fractional diffusion models	Numerical modeling	Gentamicin release from hydrogels
7	Noor et al.^41^	2024	Fractional block method	New fractional block method	Three-compartment pharmacokinetics
8	Li & Jusko^42^	2023	Minimal physiologically-based PK models	Series-compartment model (SCM)	Fractional distribution assessment
9	Zaitri et al.^43^	2023	$\psi$-Caputo fractional derivatives	Picard iterative process	PK/PD anesthesia modeling
10	Miskovic-Stankovic & Atanackovic^44^	2023	General fractional derivatives	Mathematical analysis	Diffusion theory applications
11	Morales-Delgado et al.^45^	2023	Multivariate Mittag-Leffler functions	Exact analytical solutions	Fractional pharmacokinetic models
12	Kaikousidis & Dokoumetzidis^46^	2023	Fractional differential equations	NONMEM extension (FORTRAN)	Diazepam clinical data analysis
13	Khan et al.^47^	2023	fractional-order differential equations	Legendre spectral method	Numerical analysis applications
14	Mtshali & Jacobs^48^	2023	Two-compartment fractional model with Michaelis-Menten kinetics	Grunwald-Letnikov and L1 approximation	Clinical data validation
15	Miskovic-Stankovic et al.^49^	2023	Two-compartmental fractional derivative model	General fractional derivative approach	Theoretical pharmacokinetics
16	Awadalla et al.^50^	2023	Caputo-Fabrizio and Caputo fractional derivatives	Numerical methods	Drug concentration modeling
17	Noor et al.^51^	2022	One-directional fractional pharmacokinetics	Numerical simulations	Theoretical modeling
18	Mohammadi-Firouzjaei et al.^52^	2022	Fractional compartmental model	Local discontinuous Galerkin method	Pharmacokinetic applications
19	Yang et al.^53^	2022	Population pharmacokinetic model	NONMEM modeling	TQ-B3101 in pediatric patients
20	Coman & Iosif^54^	2022	PK/PD control models	MATLAB-based tool	Anesthesia control systems

A comprehensive literature search was conducted using the ISI Web of Science database to systematically analyze advancements in fractional and fractal pharmacokinetic modeling. The search was initially performed using the keywords “pharmacokinetic modeling” and “fractional-order”, giving a total of 20 results for the period between 2022–2025. The literature, as shown in Table 2 shows substantial progress in fractional-order PK modeling, with key advances across theory, computation, and clinical application^15^. Borkor et al.^35^ conducted a comprehensive study on three types of fractional models applied to amiodarone diffusion. Their results confirmed the superiority of fractional approaches over classical models in capturing anomalous drug kinetics. In terms of software development, Kaikousidis and Dokoumetzidis^46^ introduced the first extension of NONMEM for fractional differential equations in nonlinear mixed-effects modeling. They validated the tool using clinical diazepam data, addressing a major limitation in the practical implementation of fractional PK models. Methodological innovations are also emerging. Zaitri et al.^43^ applied $\psi$-Caputo fractional derivatives to PK/PD anesthesia models, using Picard iteration to simulate drug distribution during induction. Mtshali and Jacobs^48^ provided clinical validation by showing that fractional-order models with Michaelis-Menten kinetics better describe certain drug regimes than standard models. Their parameter estimation naturally favored fractional clearance terms. Theoretical contributions include exact solutions using multivariate Mittag-Leffler functions by Morales-Delgado et al.^45^, and stochastic simulation methods for fractional models by Xu et al.^39^. Together, these developments show that fractional-order PK modeling is evolving into a practical and clinically relevant framework, especially for drugs with memory effects and non-classical kinetics.

The relevance of memory-based modeling extends beyond pharmacokinetics, as recent interdisciplinary studies have demonstrated. Olayiwola et al.^55^ used the Atangana–Baleanu–Caputo derivative to model behavioral memory in diabetes management, showing how historical exposure to education alters future health behavior. This illustrates a parallel with pharmacokinetic memory, where prior drug exposure continues to influence concentration profiles. Similarly, Dasgupta et al.^56^ introduced a fractional memory structure into a demand forecasting model, proving that long-memory dynamics enhance system responsiveness to historical stimuli. These studies reinforce the core assumption behind the models presented in this paper: that the physiological system (here, adipose tissue) exhibits non-local temporal behavior best captured via fractional operators.

Beyond phenomenological modeling, Zouari et al.^57^ embedded fractional-order pharmacodynamics within an adaptive backstepping neural control framework for chemotherapy. The memory property in their model allowed the control algorithm to implicitly account for drug accumulation and delayed tumor response. Their results showed superior performance over integer-order formulations, underscoring the practical utility of fractional models in control contexts. Analogously, our framework offers a potential foundation for closed-loop anesthesia where delayed drug redistribution (e.g., from fat stores) critically affects drug effect prediction.

Finally, the work of Pai^58^ highlights a major gap in current dosing practice for obese patients. Standard scaling approaches based on body weight or surface area fail to account for the disproportionate drug distribution into adipose compartments, leading to suboptimal exposure. Our model directly addresses this limitation by introducing both a memory-aware formulation (fractional-order) and a physiologically motivated trap compartment. In contrast to the empirical or static assumptions in classical models, our approach provides a dynamic structure that adapts to the patient’s dosing history, physiological composition, and drug-specific properties.

### Digital twins use in healthcare and inpatient generators (Anesthesia/ICU)

DTs have emerged as a transformative technology in healthcare, offering real-time, patient-specific modeling to optimize clinical decisions^59^. Initially rooted in engineering, DTs represent virtual counterparts of physical entities, leveraging real-time data to simulate, monitor, and predict outcomes in critical care environments, including anesthesia and the ICU^60^. According to Lonsdale et al.^61^, the perioperative human digital twin integrates multimodal data streams, including biomarkers, electronic health records, and Internet of Medical Things devices, to enable real-time AI-driven decision-making during surgical procedures. Additionally, Alazab et al.^62^ discuss how DTs, enhanced by machine learning and graph-based modeling, address challenges like interpatient variability and resource optimization in ICUs. DTs are also pivotal for preemptive care. For example, real-time simulations can predict complications, enabling interventions before adverse events occur. This predictive capability, as highlighted by Boulos and Zhang^63^, transforms traditional reactive care into proactive management, providing a holistic view of a patient’s physiology and its trajectory under varying treatment plans. In addition to their role in clinical applications, DTs serve as rich data generators, facilitating the development, validation, and continuous refinement of new computational models and methods.

## Methods

### Pharmacokinetics

Adipose tissue is structurally distinct from other soft tissues: it is composed of large lipid-filled adipocytes embedded within a non-uniform matrix of interstitial fluid and connective tissue. This architecture results in high porosity, providing ample storage space, but low permeability, which restricts drug mobility through the tissue^64^. Moreover, adipose tissue is poorly perfused, receiving only about 5% of cardiac output, making it a slow-equilibrating and clearance-limited compartment. In obesity, these physiological limitations are further exacerbated: adipocyte hypertrophy and hyperplasia increase storage capacity, while extracellular matrix remodeling and capillary rarefaction reduce permeability and perfusion even more^65,66^. Lipophilic drugs are particularly affected. They diffuse passively into adipocytes and become sequestered within lipid droplets, leading to prolonged retention and delayed release well after dosing ends^2,22^.

To represent this delayed drug release, we adopt two complementary modeling strategies. The first is an augmented compartmental model that introduces a “trap” subcompartment within the fat compartment. In this formulation, the drug flows from fat to trap and then back to fat before re-entering the bloodstream, introducing a physiologically interpretable delay that mimics slow redistribution. Although biologically simplified, this structure captures the essential retention dynamics observed in adipose tissue. The mathematical formulation and interpretation of this delay will be detailed in the next subsection.

**Fig. 1:**
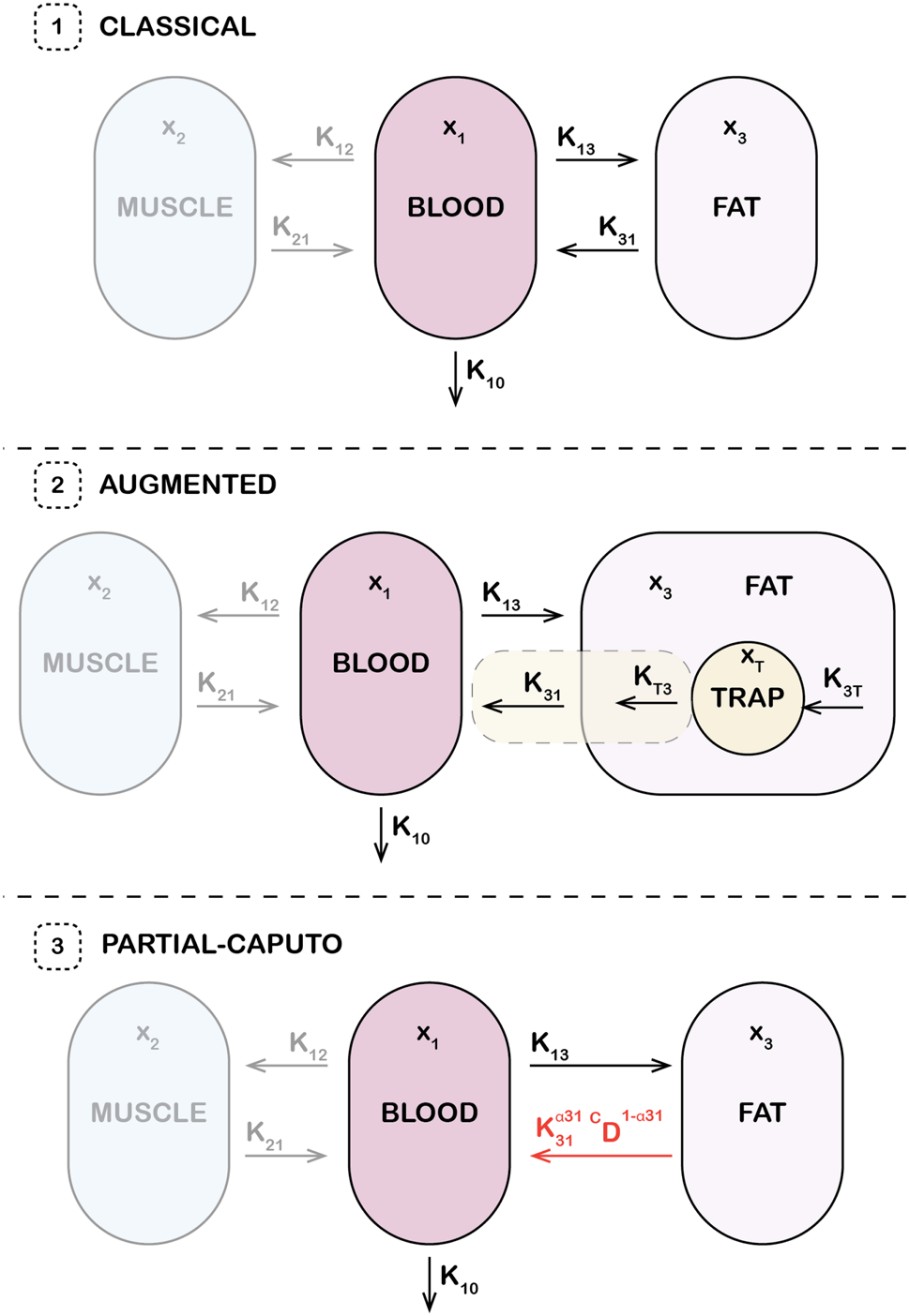
Comparative illustration of the classical PK model, augmented model, and fractional-order model.

Although the term “trap” is used in this work to describe the slow and prolonged retention of drugs within adipose tissue, we acknowledge that this usage departs from the formal definitions established in compartmental theory. Fife showed that a linear compartmental system contains a trap if and only if the system matrix has a zero eigenvalue, meaning material can enter the trap but cannot leave it^67^. Foster and Jacquez extended this by demonstrating that the multiplicity of the zero eigenvalue corresponds to the number of irreducible trap compartments within the system^68^. Jacquez and Simon defined traps as compartments, or groups of compartments, with no outflows to the environment or to other parts of the system. They emphasized that such structures contribute to structural retention and can lead to dynamic trapping behavior within compartmental models^69^. In our augmented model, however, the so-called “trap” compartment allows reversible exchange with the fat compartment (via the parameters $k_{3t}$ and $k_{t3}$, as shown in Fig. 1), and therefore behaves functionally as a lag or slow-redistribution compartment rather than a strict trap^70^. We use the term “trap” here in a broader, physiological sense, to reflect the temporary sequestration of drugs within poorly perfused adipose tissue.

Alternatively, we consider a fractional-order model in which only the fat-to-blood transition is governed by a Caputo derivative. This approach does not explicitly represent the underlying cause of drug retention but instead embeds its effects directly through memory-based dynamics. It provides a compact, phenomenological representation of long-term retention, and its implementation will also be presented in the following subsections. Figure 1 illustrates the structural differences between the classical, augmented, and fractional-order models, emphasizing how each approach handles delayed drug dynamics and retention.

#### Augmented model

In general anesthesia, the drugs are administered intravenously into the patient. The drugs then move from the central compartment i.e. blood to the peripherical compartments i.e. muscle and fat^71^. The classical three-compartmental model is given by the set of equations in (1).

1 $$\begin{aligned} \begin{aligned}&\dot{x}_1(t)=k_{21}x_2(t)-k_{12}x_1(t) +k_{31}x_3(t)-k_{13}x_1(t) -k_{10}x_1(t)+ \frac{u(t)}{V_1},\\&\dot{x}_2(t)=k_{12}x_1(t)-k_{21}x_2(t),\\&\dot{x}_3(t)=k_{13}x_1(t)-k_{31}x_3(t),\\ \end{aligned} \end{aligned}$$

where *u*(*t*), in [mass]/[time] unit, is the input infusion rate of the drug, $x_i$ for $i=1,2,3$ is the concentrations of blood, muscle, and fat respectively in [mass]/[volume] unit. The parameters $k_{ij}$, in 1/[time] unit, for $i \ne j$ are the drug transfer rates from the $i^{th}$ to the $j^{th}$ compartment, and are defined as:

2 $$\begin{aligned} \begin{array}{ll} & k_{10}=\frac{C_{l1}}{V_1}, \hspace{0.4cm} k_{12}=\frac{C_{l2}}{V_1},\hspace{0.4cm} k_{13}=\frac{C_{l3}}{V_1},\hspace{0.4cm} k_{21}=\frac{C_{l2}}{V_2}, \hspace{0.4cm} k_{31}=\frac{C_{l3}}{V_3}, \\ \end{array} \end{aligned}$$

where $C_{li}$ and $V_{i}$ for $i=1,2,3$, represent the clearance rate in [volume]/[time] and the volumes in [volume] units of the three compartments. The calculation of these parameters depends on the type of drug.

**Fig. 2:**
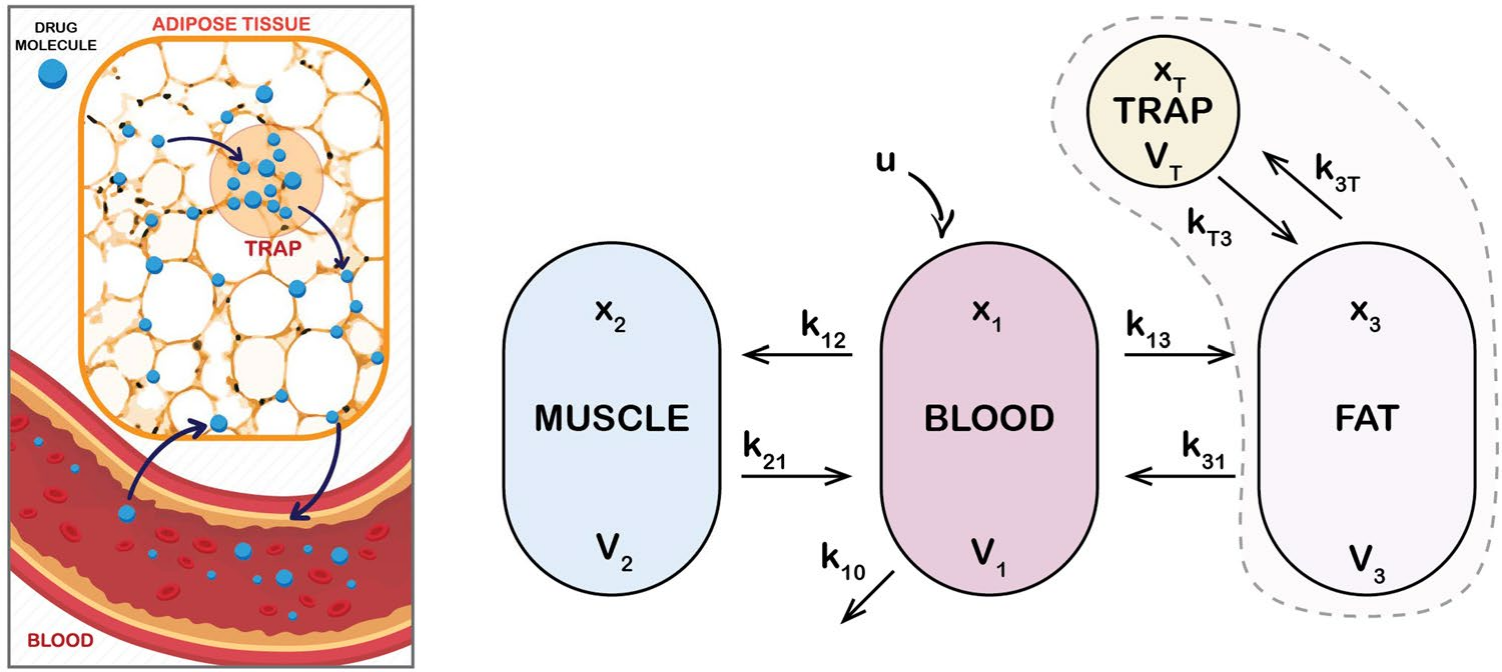
Schematic representation of the augmented pharmacokinetic model with a trapping compartment. Left: Conceptual illustration of drug transport and retention in adipose tissue. Drug molecules (blue) migrate from the bloodstream into fat, where they may become transiently trapped, representing delayed release due to limited perfusion and structural barriers within the tissue. Right: Compartmental model including blood ($x_1$, $V_1$), muscle ($x_2$, $V_2$), fat ($x_3$, $V_3$), and an additional trap compartment ($x_T$, $V_T$) with the exchange rates $k_{3T}$ and $k_{T3}$. The dashed region highlights the focus of the mathematical analysis.

The transition of a drug from one compartment to another is based on homogeneous assumptions. However, adipose tissue exhibits a complex structure and nonlinear diffusion properties in the fat compartment. As the patient’s BMI increases, fat cells aggregate into white fat, forming barriers that hinder molecular diffusion. This results in an increased duration of drug retention in fat and a decreased clearance rate. This assumption was previously introduced in^19–21^, where the model represented in Eq. (1) was expanded by introducing an additional compartment to account for drug trapping in adipose tissue as shown in Fig. 2. This model addresses limitations in classical approaches, particularly in accounting for the variations in fat distribution and its impact on drug dynamics.

To maintain tractability, several simplifying assumptions were made in the development of the augmented model. First, drug transfer between compartments is modeled using linear kinetics, assuming constant rate coefficients that do not vary with time, concentration, or physiological state. Second, each compartment is treated as spatially homogeneous, neglecting potential intra-compartment gradients or structural barriers that may influence diffusion, especially within adipose tissue. Third, tissue perfusion is assumed to be constant over time, disregarding the hemodynamic changes (e.g., due to anesthesia or comorbidities) that could alter drug delivery and clearance. Fourth, the model does not explicitly account for passive diffusion mechanisms driven by the drug’s lipophilicity, which can significantly influence distribution into and out of adipose tissue. While these assumptions simplify the analysis and facilitate numerical simulation, they may limit the model’s ability to fully capture dynamic or heterogeneous physiological processes.

The trap-compartment equation is formulated using standard mass balance principles. It assumes that drug enters the trap compartment from the fat compartment at a rate proportional to its concentration, and exits back at a rate proportional to its own concentration as shown in Fig. 2.It is represented by:

3 $$\begin{aligned} \dot{x}_t(t)=k_{3t}x_3(t)-k_{t3}x_t(t), \end{aligned}$$

where $x_{t}$ represents the concentration in the trap compartment, $k_{3t}$, in 1/[Time], is the drug transfer coefficient between the fat compartment and the fat trap compartment. $k_{t3}$, also in 1/[Time], represents the transfer rate from the trap compartment back into the blood and fat compartments. They are calculated as:

4 $$\begin{aligned} \begin{array}{l} k_{3t}={\frac{C_{lt}}{V_t}}, \hspace{0.5cm} k_{t3}={\frac{C_{lt}}{V_3}}, \end{array} \end{aligned}$$

with its volume and clearance rate:

5 $$\begin{aligned} V_t=V_3 \cdot BMI/ 100, \hspace{0.4cm}C_{lt}=C_{l3}/R. \end{aligned}$$

Here, $BMI = \frac{W}{H^2}$ denotes the body mass index, expressed in kg/$\hbox {m}^2$, where *W* is the body weight in kilograms and *H* is the height in meters. *R* may be considered as the risk for trapping which was defined in^72^. In this work, the risk data was fitted using both linear and nonlinear regression models. For the nonlinear case, the data was approximated using a sum-of-sines model with different numbers of sine components *m*, which varies from 3 to 6 terms. The parameters $\theta = [a_1, b_1, c_1, \ldots , a_m, b_m, c_m]$ were estimated using the Trust-Region algorithm, an iterative optimization method designed to minimize the sum of squared residuals in a bounded region around the current estimate. To evaluate the model performance and select the best trade-off between accuracy and complexity, several criteria were used. Among all tested models, the 3-sine model was the best at achieving the lowest Corrected Akaike Information Criterion value. The risk term *R* was approximated using a sum-of-sines function because this flexible structure is well-suited for capturing complex, nonlinear trends^72^. It provides a smooth and continuous approximation, while ensuring a good fit across the entire BMI range. It is represented as:

6 $$\begin{aligned} R=\sum _{i=1}^{3}{a_i \cdot \sin (b_i \cdot \text {BMI} + c_i)}. \end{aligned}$$

The parameters of this equation are given in Table 3.

**Table 3:** Parameters of the R equation.

$a_1$	$b_1$	$c_1$	$a_2$	$b_2$	$c_2$	$a_3$	$b_3$	$c_3$
27.4472	0.0033	−0.0570	0.8002	0.5390	−4.2180	0.4815	0.7595	−6.2212

With the introduction of the trap compartment, the set of equations in (1) becomes:

7a $$\begin{aligned}&\dot{x}_1(t)=-k_{12}x_1(t)-k_{13}x_1(t)-k_{10}x_1(t)+k_{21}x_2(t) +k_{31}x_3(t)+ \frac{u(t)}{V_1}, \end{aligned}$$

7b $$\begin{aligned}&\dot{x}_2(t) = k_{12}x_1(t) - k_{21}x_2(t), \end{aligned}$$

7c $$\begin{aligned}&\dot{x}_3(t)=k_{13}x_1(t)-k_{31}x_3(t)-k_{3t}x_3(t)+k_{t3}x_t(t), \end{aligned}$$

7d $$\begin{aligned}&\dot{x}_t(t)=k_{3t}x_3(t)- k_{t3}x_t(t). \end{aligned}$$

To understand the dynamic effect of the trap compartment on the system’s memory, we analyze its contribution using a numerical scheme.

The purpose of the next part is to show that adding a compartment, in this case the trap compartment, will add a memory effect to the fat compartment. To do so, we apply the forward Euler discretization to the differential equation describing the trap compartment (7d), transforming it into the discrete-time form shown in Eq. (8). The Euler method is one of the simplest numerical techniques for solving ordinary differential equations (ODEs). By discretizing the independent variable, it transforms ODEs into algebraic equations, allowing efficient computation^73^. To solve the differential equation in (3), The forward Euler method was used. The result is then :

8 $$\begin{aligned} \frac{x_{t}(n)- x_{t}(n-1)}{h} = k_{3t} x_{3}(n-1) - k_{t3} x_{t}(n-1), \end{aligned}$$

for $n \in \{1, 2, \ldots , N\}$ and the initial condition $x_{t}(0)$ and $x_{3}(0)$. The discrete-time variable $x_t(n)$ denotes the value of the numerically computed solution at time $t = nh$, with *h* representing the step size and *n* the time index. In the forward Euler method, the derivative $\dot{x}_t(t)$ is replaced by a first-order finite difference, resulting in the update Eq. (8). This discretization transforms the differential Eq. (3) into an iterative algebraic form. The accuracy of the solution depends on the choice of *h*: smaller values lead to a closer alignment between $x_t(n)$ and the continuous-time trajectory $x_t(t)$, while larger values may result in discretization errors and numerical instability. For $n=1$, Eq (8) is:

9 $$\begin{aligned} x_{t}(1) = (1-k_{t3}h ) x_{t}(0) + (k_{3t}h) x_{3}(0). \end{aligned}$$

For simplification, we put $A=(1-k_{t3}h )$ and $B=(k_{3t}h)$. The next step is to calculate $x_{t}$ until the $n^{th}$ term, where the total simulation time $T=hN$:

10 $$\begin{aligned} \begin{aligned} x_t(2)&= A \big (A x_t(0) + B x_3(0)\big ) + B x_3(1) \\&= A^2 x_t(0) + AB x_3(0) + B x_3(1) \\ x_t(3)&= A \big (A^2 x_t(0) + AB x_3(0) + B x_3(1)\big ) + B x_3(2) \\&= A^3 x_t(0) + A^2 B x_3(0) + AB x_3(1) + B x_3(2) \\&\vdots \\ x_t(n)&= A^n x_t(0) + B(A^{n-1} x_3(0) + A^{n-2} x_3(1) + \ldots + A^{0} x_3(n-1))\\&= A^n x_t(0) + B \sum _{i=1}^{n} A^{i-1} x_3(n-i)\\&\vdots \\ x_t(N)&= A^n x_t(0) + B \sum _{i=1}^{N} A^{i-1} x_3(N-i). \end{aligned} \end{aligned}$$

Before surgery, no drug is present in the body, i.e., $x_t(0) = 0$ and $x_3(0) = 0$. Consequently, the term $A^n x_t(0)=0$, and the final expression becomes:

11 $$\begin{aligned} x_t(n) = k_{3t}h \sum _{i=1}^{n} (1-k_{t3}h )^{i-1} x_3(n-i). \end{aligned}$$

By applying the forward Euler method to the differential equation (7c), and substituting the expression for $x_t(n-1)$ from Eq. (11), leads to:

12 $$\begin{aligned} \begin{aligned} \frac{x_{3}(n) - x_{3}(n-1)}{h}&= k_{13} x_{1}(n-1)- k_{31} x_{3}(n-1) \\&- k_{3t} x_{3}(n-1) + k_{t3} \left[ k_{3t}h \sum _{i=1}^{n-1} (1-k_{t3}h )^{i-1} x_3(n-1-i)\right] \\&= k_{13} x_{1}(n-1) - k_{31} x_{3}(n-1) \\&- k_{3t} \left[ x_{3}(n-1) - \sum _{i=1}^{n-1} k_{t3}h (1-k_{t3}h )^{i-1} x_3(n-1-i)\right] .\\ \end{aligned} \end{aligned}$$

From Eq. (12), the term $k_{t3}h (1-k_{t3}h )^{i-1}$ can be further developed using the binomial expansion formula (see Appendix A), which leads to:

13 $$\begin{aligned} \begin{aligned}&\frac{x_{3}(n) - x_{3}(n-1)}{h} = k_{13} x_{1}(n-1) - k_{31} x_{3}(n-1) \\&-k_{3t} \left[ x_{3}(n-1) - \sum _{i=1}^{n-1} \left( \sum _{k=1}^{i} \left( {\begin{array}{c}i-1\\ k-1\end{array}}\right) (-1)^{k-1} (k_{t3}h)^{k}\right) x_3(n-1-i)\right] . \end{aligned} \end{aligned}$$

The full derivation from Eq. (12) to Eq. (13) is provided in Appendix A.

As shown through the discretization of Eq. (7c) into Eq. (13), the introduction of the trap compartment leads to a recursive structure in the dynamics of the fat compartment. The summation term in Eq. (13) introduces a memory effect, where $x_3(n)$ is influenced not only by its immediate past state but also by a weighted sum of its previous values. The nested summations involving binomial coefficients and powers of $k_t h$ in

$$\begin{aligned} k_{t3}h(1 -k_{t3}h)^{i-1} = \sum _{k=1}^{i} \left( {\begin{array}{c}i-1\\ k-1\end{array}}\right) (-1)^{k-1} (k_{t3}h)^{k}. \end{aligned}$$

act as a memory kernel, delaying the response of $x_3$ by spreading the effect across multiple time steps.

This behavior is analogous to phenomena such as anomalous diffusion or fractional-order kinetics, where systems display history-dependent dynamics. The inclusion of the trap compartment thus imparts both historical dependence and non-instantaneous response to $x_3$, which are key characteristics of systems with memory effects and biological processes with retention effects.

The parameter $0< k_{t3} h < 1$ introduces a fading memory effect in the dynamics of $x_3(n)$. In particular, past values of $x_3$ appear in the model weighted by the factor $(1 - k_{t3} h)^{i-1}$, which decreases with increasing *i*. This means that more recent past states contribute more strongly to the current value, while older states have less influence. The speed at which this influence fades is controlled by $k_{t3} h$: when $k_{t3} h$ is small, the decay is slow and the system retains memory of past values for a longer time; when $k_{t3} h$ is large, the decay is faster and the memory becomes short-term. Consequently, $k_{t3} h$ acts as a tuning parameter that determines how quickly the influence of past states diminishes over time.

Although the memory kernel is exponential rather than power-law, its structure is qualitatively reminiscent of fractional-order systems. In fractional calculus, memory effects are governed by convolution kernels with algebraic decay, which lead to nonlocal operators like the Caputo or Riemann–Liouville derivatives. These fractional models possess long memory, where the influence of the past decays slowly and never fully vanishes. While the kernel in Eq. (13) lacks the power-law structure and infinite memory of true fractional operators, it nonetheless captures key features associated with fractional-order behavior: history dependence, delayed response, and finite-memory effects. In this sense, the exponential kernel provides a simplified representation of memory effects similar to those found in fractional-order systems, offering a biologically meaningful structure within a standard compartmental modeling framework. In the context of drug dynamics, this fading memory represents the gradual clearance and decreasing influence of the drug stored in fat, highlighting the importance of accounting for retention effects in PK models.

#### Fractional-order compartmental modeling

Fractional-order compartmental models extend classical approaches by replacing integer-order derivatives with derivatives of non-integer order^74^. This generalization enables the system to exhibit memory effects, where the future state depends not only on the present but also on the entire history of the system’s evolution. Such behavior is particularly relevant in biomedical applications, where processes like drug retention, tissue diffusion, and delayed exchange often deviate from ideal first-order kinetics^75,76^. Among the different definitions in fractional calculus, the Caputo and Riemann–Liouville (RL) derivatives are the most commonly used due to their well-established mathematical properties and applicability to physical systems.

The Caputo derivative is widely adopted in applied modeling because it allows the use of standard initial conditions and is defined as:

14 $$\begin{aligned} {}^C D_t^\alpha x(t) = ^{RL}{I}^{1-\alpha } \dot{x}(t) = \frac{1}{\Gamma (1-\alpha )} \int _0^t \frac{\dot{x}(\tau )}{(t-\tau )^\alpha } \, d\tau , \end{aligned}$$

where $\alpha \in (0,1)$ is the fractional-order, t is the current time, $\tau$ is the integration variable representing past time, $\Gamma (\cdot )$ represents the gamma function, and ${}^{RL}{I}$ is the Riemann-Liouville integral which is defined by:

15 $$\begin{aligned} ^{RL}I^{\alpha }x(t) = \frac{1}{\Gamma (\alpha )} \int _{0}^{t} (t - \tau )^{\alpha - 1} x(\tau ) \, d\tau . \end{aligned}$$

By contrast, the RL derivative requires initial conditions involving fractional integrals, which are difficult to measure experimentally^77^. While both formulations capture memory effects, Caputo is generally favored in simulation and real-world modeling due to its compatibility with classical initial conditions^78–80^. Both definitions are equivalent when the initial conditions are zero . In such cases, the choice between Caputo and RL becomes one of convenience or context.

The Caputo formulation is particularly well suited for modeling systems that exhibit compartment-specific memory effects. This is especially important in heterogeneous systems, where some compartments show long-term memory effects while others do not. As discussed in Calatayud *et al.*^15^, applying a fractional derivative selectively to specific transitions enables a more realistic representation of asymmetric dynamics. This “partial Caputo” approach preserves the physical structure of fluxes and mass conservation laws, while allowing different compartments or pathways to express different memory behaviors. Moreover, this selective memory structure has a natural interpretation in terms of survival analysis: the Caputo derivative induces a time-dependent hazard function, meaning that the longer a substance remains in a given compartment, the lower its instantaneous probability of leaving. This history-dependent behavior reflects physiological processes such as drug entrapment in adipose tissue, where prolonged retention leads to progressively slower release rates.

A representative example of compartment-specific memory can be found in the modeling of anomalous transport through heterogeneous media. In porous or biological environments, such as layered tissues, polymer networks, or cellular membranes, certain compartments significantly delay particle transit due to trapping, obstruction, or binding interactions, while others allow faster passage. This creates a system where memory effects arise only in select compartments. In these contexts, fractional models using Caputo derivatives have been employed to describe long retention times by assigning non-integer dynamics only to those compartments that exhibit subdiffusion or prolonged storage^81,82^. For instance, a two-compartment model might apply an integer-order derivative to a fast-clearing vascular region and a Caputo derivative with $\alpha < 1$ to a compartment representing tissue entrapment or slow exchange. The Caputo kernel naturally accounts for the full history of occupancy in that compartment. This leads to a decreasing transition probability over time, which aligns with observed slow-release or trapping behavior. The resulting solution captures long-term memory via a Mittag-Leffler decay, distinguishing compartments that accumulate and retain material from those that respond quickly. By allowing $\alpha$ to vary by compartment or flux, the Caputo approach enables a physiologically meaningful representation of heterogeneous retention, while preserving the overall mass balance and interpretability of the model.

This approach is supported physiologically. The characteristic time constants of the compartments vary significantly: approximately 10–12 min for blood, 20–30 min for muscle, and 2–3 h for fat^83^. Moreover, drug uptake and release from fat differ mechanistically: while lipophilic drugs such as Propofol accumulate rapidly^3^, their release is typically slower. Modeling this asymmetry using a uniform fractional-order would be unrealistic. Other formulations such as Grünwald–Letnikov require a uniform fractional-order across all compartments to preserve mass balance, which contradicts the degree of heterogeneity observed in many physiological and social systems^17,84^. Similarly, exponential-memory models (e.g., Caputo–Fabrizio, Atangana–Baleanu) decay too quickly to capture long-tailed retention behavior and generally underestimate the time a state persists. For these reasons, we use the Caputo derivative to model only the flux that exhibits long-term memory, while retaining integer-order dynamics elsewhere.

To illustrate this idea, we consider the classical first-order elimination process:

16 $$\begin{aligned} \dot{x}(t) = -K x(t), \end{aligned}$$

and its fractional analog:

17 $$\begin{aligned} {}^C D_t^\alpha x(t) = -K^\alpha x(t). \end{aligned}$$

Under zero initial conditions, this Caputo model is equivalent to an RL formulation involving a convolution:

18 $$\begin{aligned} \dot{x}(t) = -K^\alpha \; {}^{RL}D^{1-\alpha } x(t), \end{aligned}$$

where

19 $$\begin{aligned} {}^{RL}D^{1-\alpha } x(t) = \frac{1}{\Gamma (\alpha )} \frac{d}{dt} \int _0^t \frac{x(\tau )}{(t - \tau )^{1-\alpha }} d\tau . \end{aligned}$$

We used this equivalence to express the final model in terms of RL derivatives for notational consistency and numerical implementation. The full Caputo formulation applied to all compartments reads:

20 $$\begin{aligned} \begin{aligned} {}^C D_t^\alpha x_1&= k_{21}^\alpha x_2 + k_{31}^\alpha x_3 - (k_{12}^\alpha + k_{13}^\alpha + k_{10}^\alpha ) x_1 + \frac{u(t)}{V_1}, \\ {}^C D_t^\alpha x_2&= k_{12}^\alpha x_1 - k_{21}^\alpha x_2, \\ {}^C D_t^\alpha x_3&= k_{13}^\alpha x_1 - k_{31}^\alpha x_3, \end{aligned} \end{aligned}$$

but assumes homogeneous memory behavior, which is not physiologically realistic.

To address this, we adopted a partial-fractional model that preserves mass balance while allowing each flux to have its own memory exponent:

21 $$\begin{aligned} \begin{aligned} \dot{x}_1(t)&=k_{21}^{\alpha _{21}}\;{}^{RL}D^{1-\alpha _{21}}x_2(t)+k_{31}^{\alpha _{31}}\;{}^{RL}D^{1-\alpha _{31}}x_3(t)-k_{12}^{\alpha _{12}}\;{}^{RL}D^{1-\alpha _{12}}x_1(t) \\&\quad -k_{13}^{\alpha _{13}}\;{}^{RL}D^{1-\alpha _{13}}x_1(t) -k_{10}^{\alpha _{10}}\;{}^{RL}D^{1-\alpha _{10}}x_1(t) + \frac{u(t)}{V_1},\\ \dot{x}_2(t)&=k_{12}^{\alpha _{12}}\;{}^{RL}D^{1-\alpha _{12}}x_1(t)-k_{21}^{\alpha _{21}}\;{}^{RL}D^{1-\alpha _{21}}x_2(t),\\ \dot{x}_3(t)&=k_{13}^{\alpha _{13}}\;{}^{RL}D^{1-\alpha _{13}}x_1(t)-k_{31}^{\alpha _{31}}\;{}^{RL}D^{1-\alpha _{31}}x_3(t). \end{aligned} \end{aligned}$$

where $\alpha _{ij}$ denotes the fractional-order for each transition.

In our model, only the release from fat to blood was modeled with $\alpha _{31} < 1$, while all other transitions were modeled with $\alpha _{12} = \alpha _{21} =\alpha _{13} = \alpha _{10} = 1$. This design allowed us to capture subdiffusive behavior in the fat compartment, in line with experimental expectations, while preserving classical kinetics elsewhere. Although this formulation preserves mass balance, its mathematical well-posedness still requires formal verification^15^.

To solve the equations in (21), the Laplace transform remains theoretically applicable if specific conditions are satisfied. In particular, to ensure Laplace transform convergence, the input *u*(*t*) (drug infusion rate) must satisfy $|u(t)| \le Me^{ct}$, where $M, c > 0$, ensuring that the integral $\int _0^{\infty } e^{-st} u(t) \, dt$ converges for $\Re (s) > c$^85^. Common anesthesia inputs like step functions (bolus) or rectangular pulses (infusion) meet this criterion. The second condition is that all RL fractional derivatives must have zero initial memory (i.e. $x_i(0) = 0$). As explained earlier, this condition is also met. Under these assumptions, Laplace transformation gives an algebraic system where fractional differentiation appears as $s^{1-\alpha _{ij}}$ in the Laplace domain. This allows the equations to be rearranged into a matrix form $\textbf{A}(s)\textbf{X}(s) = \textbf{B}(s)$, which can be solved analytically for simple inputs or numerically inverted for more complex input profiles (e.g., PID-driven infusion).

Applying the Laplace transform to the system in Eq. (21) under the zero initial memory condition yields:

22 $$\begin{aligned} \begin{aligned} sX_1(s)&= k_{21}^{\alpha _{21}} s^{1-\alpha _{21}} X_2(s) + k_{31}^{\alpha _{31}} s^{1-\alpha _{31}} X_3(s) \\&\quad - \left( k_{12}^{\alpha _{12}} s^{1-\alpha _{12}} + k_{13}^{\alpha _{13}} s^{1-\alpha _{13}} + k_{10}^{\alpha _{10}} s^{1-\alpha _{10}} \right) X_1(s) + \frac{U(s)}{V_1}, \\ sX_2(s)&= k_{12}^{\alpha _{12}} s^{1-\alpha _{12}} X_1(s) - k_{21}^{\alpha _{21}} s^{1-\alpha _{21}} X_2(s), \\ sX_3(s)&= k_{13}^{\alpha _{13}} s^{1-\alpha _{13}} X_1(s) - k_{31}^{\alpha _{31}} s^{1-\alpha _{31}} X_3(s). \end{aligned} \end{aligned}$$

These equations can be rearranged into the compact matrix form:

23 $$\begin{aligned} \underbrace{ \begin{bmatrix} s + k_{12}^{\alpha _{12}} s^{1-\alpha _{12}} + k_{13}^{\alpha _{13}} s^{1-\alpha _{13}} + k_{10}^{\alpha _{10}} s^{1-\alpha _{10}} & -k_{21}^{\alpha _{21}} s^{1 - \alpha _{21}} & -k_{31}^{\alpha _{31}} s^{1 - \alpha _{31}} \\ -k_{12}^{\alpha _{12}} s^{1 - \alpha _{12}} & s + k_{21}^{\alpha _{21}} s^{1 - \alpha _{21}} & 0 \\ -k_{13}^{\alpha _{13}} s^{1 - \alpha _{13}} & 0 & s + k_{31}^{\alpha _{31}} s^{1 - \alpha _{31}} \end{bmatrix} }_{\textbf{A}(s)} \begin{bmatrix} X_1(s) \\ X_2(s) \\ X_3(s) \end{bmatrix} = \underbrace{ \begin{bmatrix} \frac{U(s)}{V_1} \\ 0 \\ 0 \end{bmatrix} }_{\textbf{B}(s)}. \end{aligned}$$

Despite this analytical convenience, the Laplace approach presents several critical limitations that restrict its use in realistic pharmacokinetic simulations in this case. In particular, the presence of different fractional orders ($\alpha _{ij} \ne \alpha$) prevents eigenvalue decomposition, complicating both theoretical analysis and controller design. This heterogeneity is expected in real-world drug diffusion and redistribution processes.

Due to these limitations, our study opted for a simple numerical time-domain solution approach. Specifically, we used a trapezoidal quadrature method to evaluate the Caputo fractional derivatives and coupled this with an Euler integration scheme to solve the state equations. This hybrid strategy enables efficient simulation even under variable infusion protocols and supports incorporation into real-time control loops.

### Pharmacodynamics

Pharmacodynamics (PD) describes the relationship between drug concentration and its effect on the body. It is typically modeled using mathematical equations that characterize how a drug interacts with its target site to produce a physiological response. In compartmental modeling, the PD model is represented by the effect-site compartment, which accounts for the time delay between plasma drug concentration, and the drug effect equation^86^. The effect-site compartment is represented by:

24 $$\begin{aligned} \dot{x}_e(t) = k_{1e} x_1(t) - k_{e0} x_e(t), \end{aligned}$$

where $k_{1e}$ represents drug transfer rates from the plasma to the effect site compartment. $k_{e0}$ is the effect-site equilibration rate constant, both in 1/[time] unit.

The drug effect is represented by:

25 $$\begin{aligned} \begin{array}{ll} E(t)=E_{0}-E_{max}\frac{I(t)^{\gamma }}{1+I(t)^{\gamma }}, \end{array} \end{aligned}$$

where $E_0$ is the baseline effect in the absence of the drug, and $E_{max}$ is the maximum achievable effect.

When one drug is infused, *I*(*t*) is calculated as:

26 $$\begin{aligned} \begin{array}{ll} I(t)=\frac{x_e(t)}{C_{50}}, \end{array} \end{aligned}$$

where $C_{50}$ [mass]/[volume] is the concentration at half-effect (50%), $\gamma _p$ [-] describes the steepness of the concentration-effect relationship. Equation 26 with 25 represents the well established Hill equation. In pharmacology, the Hill equation is adapted to analyse drug-receptor interactions and quantify the functional parameters of drugs. It is commonly used and is particularly useful for describing nonlinear dose-response relationships, where the effect of a drug is not directly proportional to its concentration^87^

When two drugs are infused together, *I*(*t*) becomes:

27 $$\begin{aligned} \begin{array}{ll} I(t)=\left( \frac{x_{e,D1}}{C_{50,D2}} + \frac{x_{e,D2}}{C_{50,D2}} + \Gamma \cdot \frac{x_{e,D1}}{C_{50,D1}} \cdot \frac{x_{e,D2}}{C_{50,D2}} \right) , \end{array} \end{aligned}$$

where the subscripts D1 and D2 denote the first and second drugs, respectively. $\Gamma$ is the interaction parameter. Equation 27 with 25 represents the Greco?type Response Surface Model (RSM). This lattert is well?established in anesthesia for characterizing synergistic, additive, or infra?additive effects between co-administered drugs. The RSM integrates traditional isobolographic analysis and concentration–effect curves into a unified surface, allowing a comprehensive assessment of drug interactions and their influence on clinical endpoints^88^.

## Results

The previous section introduced two physiologically motivated extensions of the classical PK model. This section presents the simulation results used to validate these models, focusing on their ability to simulate BIS dynamics under various anesthetic dosing protocols. In addition, we provide a comparative analysis of model accuracy, sensitivity to key parameters, and clinical relevance. To assess model performance, we focus on the depth of hypnosis (DoH) during general anesthesia, which refers to the level of sedation and unconsciousness induced in a patient. DoH is characterized by the suppression of awareness and responsiveness and is typically monitored using the BIS, a numerical scale ranging from 0 (no measurable electrical brain activity, associated with deep coma or brain death) to 100 (fully awake), derived from electroencephalogram (EEG) signals.

**Fig. 3:**
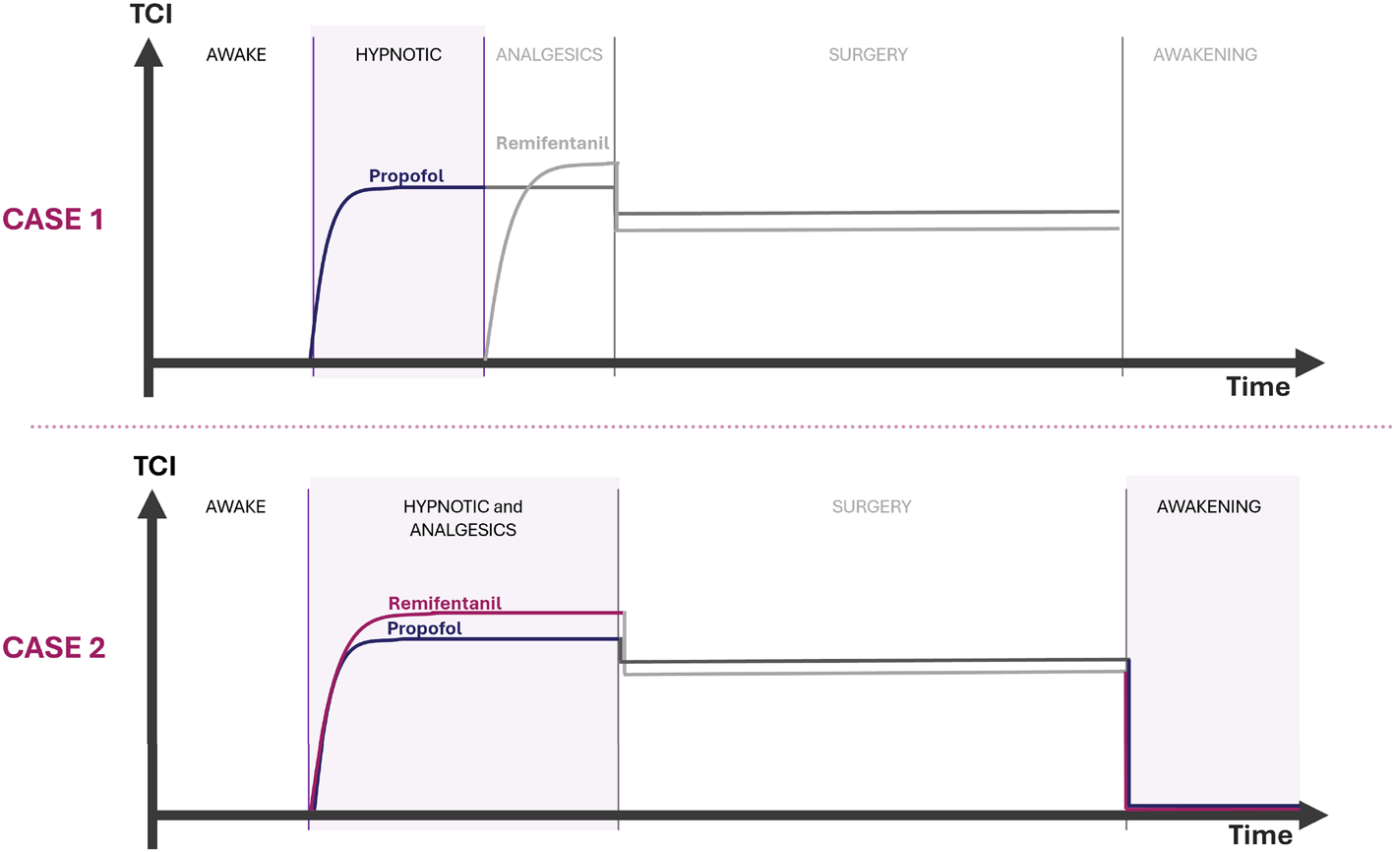
Visualization of the drug infusion protocols for case 1 and case 2. In case 1, Propofol and Remifentanil are infused separately, with the analysis focusing solely on the hypnotic region (highlighted in purple). In case 2, both drugs are administered simultaneously, enabling the study of both the hypnotic-analgesic region and the awakening region.

In anesthesia, Propofol and Remifentanil are commonly used due to their rapid onset. The focus on these two drugs was primarily motivated by the availability of high-resolution clinical data from the VitalDB database, where these two drugs were consistently administered during general anesthesia. Additionally, they are among the most commonly used agents in Western Europe for intravenous anesthesia and opioid analgesia, making them clinically relevant case studies.

From a modeling point of view, these drugs offer complementary pharmacokinetic characteristics that allow us to illustrate the concept of pharmacokinetic memory. Propofol, a highly lipophilic anesthetic with a large volume of distribution, demonstrates this effect prominently. After rapid initial redistribution from the central compartment to peripheral tissues, particularly adipose tissue, Propofol continues to exert pharmacokinetic influence through slow release from fat stores. This is further exacerbated in obese patients, where increased fat mass alters distribution and clearance dynamics. These characteristics make Propofol particularly suitable for memory-based modeling. For this reason, the pharmacokinetics of Propofol are modeled using the equations in (7). A comparison is also made using the equations in (21). The coefficients for both models are calculated based on the Schnider model presented in^71^. By contrast, Remifentanil exhibits a distinct pharmacokinetic profile. It is rapidly metabolized by nonspecific esterases in the blood and tissues, resulting in an extremely short context-sensitive half-time that is independent of infusion duration. This rapid metabolism prevents significant accumulation in tissues, allowing for a consistent and predictable offset of action, regardless of how long the drug is administered. For this reason, the pharmacokinetics of Remifentanil are modeled using the equations in (1). The model parameters are calculated using the Minto model described in^89^. Together, these two drugs provide a useful contrast between systems with and without pharmacokinetic memory. Their distinct behaviors justify their selection as representative examples for exploring memory-based models in anesthesia. A summary of commonly used anesthetic and opioid agents, including their fat accumulation tendencies, is provided in Table 1.

When Propofol is infused alone, the depth of hypnosis typically correlates with its dosage, resulting in predictable reductions in BIS values. In simulation, BIS is computed using Eq. (25) in combination with (26). When administered together with Remifentanil, Propofol exhibits a synergistic effect, allowing lower doses to achieve similar levels of hypnotic depth^90^. This combination improves anesthesia quality by optimizing hypnosis, enhancing analgesia, and promoting hemodynamic stability. In the combined case, BIS is computed using Eq. (25) with (27).

Figure 3 represents TIVA regions, namely: Awake, Hypnotic, Analgetics, Surgery, and Awakening. This section aims to analyze the effect of Propofol trapping in obese patients. To this end, two cases will be studied as shown in Fig. 3: (1) a SISO case, where Propofol is infused alone in a patient model, and (2) a MISO case, where Propofol and Remifentanil are infused together. It is important to note that the PK parameters were not estimated from data but computed using established formulas from the Schnider and Minto models (as presented earlier), based on biometrics of patients. The only parameter varied in this work is the fractional-order term, which governs the memory effect in the fat-to-blood transition. The PD parameters ($C_{50}$, $\gamma$, $\Gamma$) used in the response surface model (RSM) were previously identified using a Genetic Algorithm in our earlier study^91^, where confidence intervals were reported. In addition, it is also worth noting that all quantitative results presented in the MISO case are proof-of-concept findings based on a single high-resolution. Population-level validation is planned as part of future work.

### SISO case: propofol to BIS

#### Evaluation of drug redistribution using the proposed PK models

For this case, patient 21 from the database in^22,91^ was selected. With a BMI of 31.2, this patient is a suitable candidate for studying the effect of adipose tissue on DoH. The Biometrics of this patient is represented in Table 4, and the calculated PK parameters are presented in Table 5. The sensitivity values of this patient were estimated using the Genetic algorithm^22,92^. The analysis focuses on the hypnotic region, as shown in Fig. 3, where the patient transitions from an awake to a hypnotic state. Since only Propofol is infused, confounding factors such as drug synergy and surgical stimuli are eliminated, ensuring a controlled environment for evaluating the effect of PK models.

**Table 4:** PK model biometric values and PD model sensitivity values of patient 21.

Index	Gender	Age	Height	Weight	BMI	LBM	$C_{50}$	$\gamma$	$E_0$	Surgery
–	–	(Years)	(cm)	(kg)	(kg/m^2^)	–	(µg/ml)	–	–	–
21	F	62	168	88	31.2	54	5.6	3.7	97.7	Hysterectomy

**Table 5:** Pharmacokinetic parameters for Patient 21.

$\text {k10}$	$\text {k12}$	$\text {k13}$	$\text {k21}$	$\text {k31}$	$\text {k3t}$	$\text {kt3}$	$\text {ke0}$	$\text {k1e}$	$\text {Cl1}$	$\text {Cl2}$	$\text {Cl3}$	$\text {R}$	$\text {Clt}$	$\text {V1}$	$\text {V2}$	$\text {V3}$	$\text {Vt}$
0.5842	0.2515	0.1958	0.0698	0.0035	0.0138	0.0043	0.456	0.456	2.4945	1.074	0.836	0.8147	1.0261	4.27	15.381	238	74.256

The Propofol infusion was applied as input to the patient’s PK-PD model to simulate the BIS response. The PK-PD model was simulated using different PK equations, including the Partial-Caputo model with varying values of $\alpha _{31}$ (representing fat release) and the augmented model. Since the focus of this study is on drug release rather than absorption, $\alpha _{13}$ is fixed at 1, with its potential variation left for future work. It is worth noting that the classical PK model in Eq. (1) is a special case of the Partial-Caputo model where $\alpha _{31} = 1$.

The numerical solution of the Partial-Caputo model combines a trapezoidal quadrature for evaluating the Caputo fractional derivatives with a Euler scheme for integrating the compartmental state equations. The Euler method, although only first-order accurate, was used due to its simplicity and widespread use in early discretization of fractional-order systems^93^. Its formulation allows easy implementation and integration into control-oriented simulations, where computational constraints and real-time performance are often prioritized over high-order accuracy.

As highlighted in^93^, the Euler rule represents a basic generating function for approximating fractional operators, particularly suitable when lower computational complexity is required. While its accuracy is limited, it enables a transparent analysis of memory accumulation in fractional dynamics. This trade-off makes it a useful exploratory tool for studying compartment-specific memory behavior in pharmacokinetic systems. Importantly, the authors also demonstrate that stable discrete-time realizations can be obtained even when using low-order Euler-based approximations, provided that the approximation is tuned to the relevant frequency range and the sampling period is appropriately selected^93^.

Although the Euler method is conditionally stable, its stability can be maintained in practice by selecting a sufficiently small integration step relative to the system dynamics^93^. In our simulations, the sampling period was chosen to ensure convergence and avoid numerical artifacts.

**Fig. 4:**
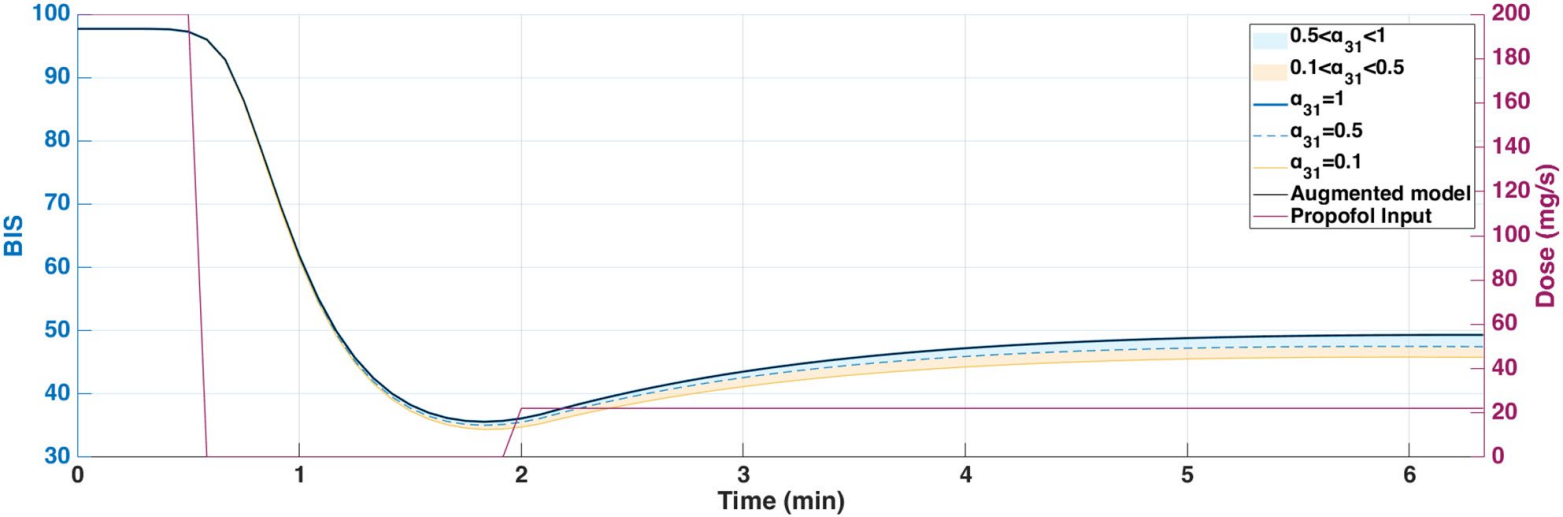
Comparison of simulated BIS responses for different values of $\alpha _{31}$ using Partial-Caputo, along with the augmented model for Patient 21.

**Fig. 5:**
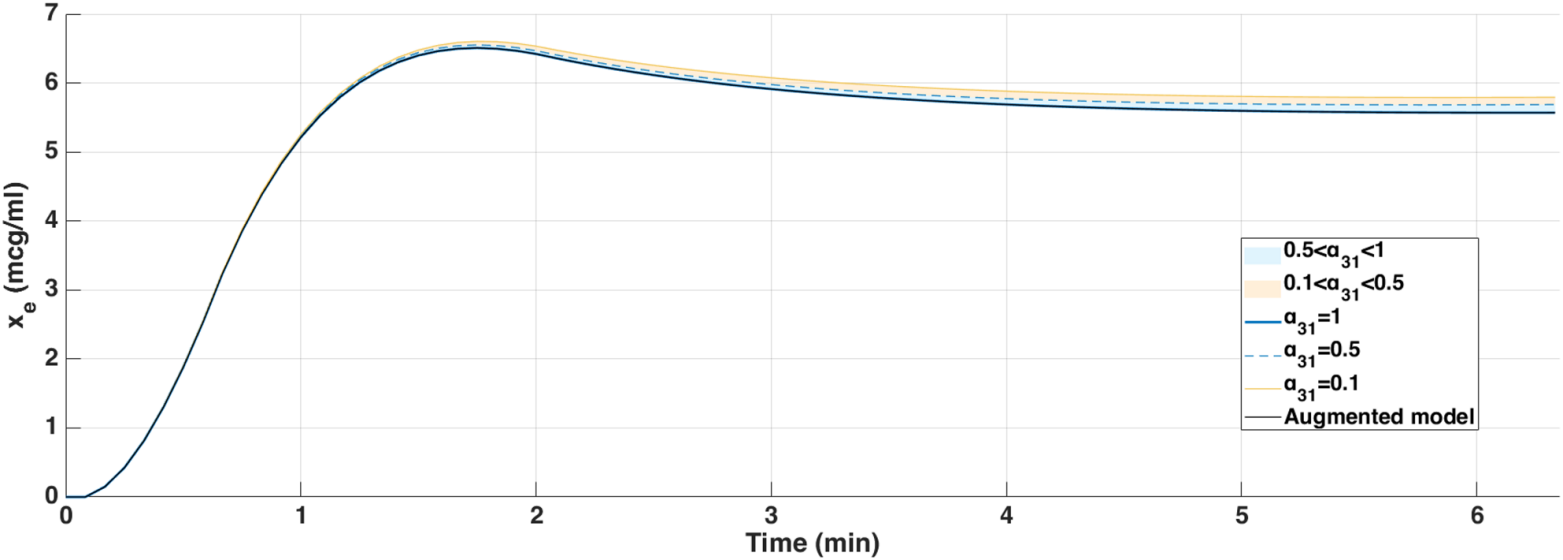
Simulated effect-site concentration ($x_e$) for different values of $\alpha _{31}$ using Partial-Caputo, along with the augmented model for Patient 21. The curves illustrate how $\alpha _{31}$ variations influence drug redistribution and effect-site kinetics.

The augmented model was also simulated with a Euler method at a sampling period of $Ts = 5s = \frac{5}{60}$ min. The parameter $\alpha _{31}$ was varied from 0.1 to 1 to study its influence on effect-site concentration and BIS output. The simulation was run for approximately *T*=6min30s corresponding to the duration of the hypnotic region. During this period, the patient’s BIS decreased from 100 to 50, the value which is generally targeted during surgical procedures. In this dosing protocol, drug administration begins with a rapid induction bolus of 120 mg delivered over 0.6 min, corresponding to a constant rate of 200 mg/min. This initial bolus is intended to increase the plasma concentration for anesthetic induction. Following this, a maintenance infusion begins at 2 min and continues until 6.4 min, delivering the drug at a rate of 230 $\mu$g/kg/min based on patient body weight. This two-phase administration profile reflects standard clinical practice, combining a fast-acting bolus with a tailored infusion to sustain the desired pharmacological effect. The results are shown in Figs.4 and 5 which represent the BIS output and the effect-site concentration respectively.

In Fig. 4, when Propofol is firstly infused (Between 0 - 1 min), all models capture the rapid drop in BIS from about 100 down to 40–50. This represents the first bolus that the anesthesiologist infuse to the patient to drag the patient from the awake state to the hypnotic state. After 2 min, where the infusion of Propofol is nearly constant to maintain the patient into deep sedation, the simulated models diverge slightly in how quickly the BIS rebounds. Larger $\alpha _{31}$ values (closer to 1), and the augmented model, behave more like the classical three-compartment PK model, whereas smaller $\alpha _{31}$ prolongs the effect slightly. The change in $\alpha _{31}$ also affects the predicted concentration of the effect site as shown in Fig. 5. In the induction phase (first bolus). All models rise to a similar peak near 6-7 $\mu$ g/mL at around 2 min and then decline (Fig. 5). The fractional-order parameter $\alpha _{31}$ alters how quickly Propofol redistributes from adipose tissue (the fat compartment). Again, the curves look very similar for $\alpha _{31}$ in the range 0.5–1.0 but deviate more if $\alpha _{31}$ is as low as 0.1.

**Fig. 6:**
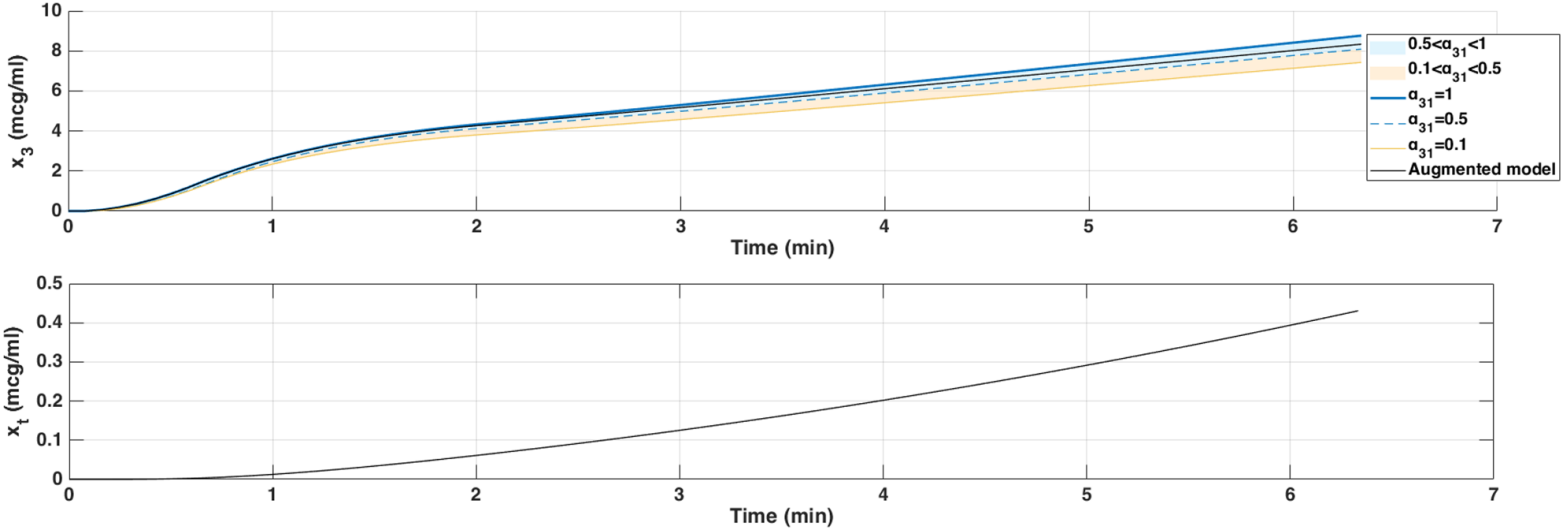
Drug concentration in the fat compartment ($x_3$, top) for different values of $\alpha _ {31}$ in the Partial-Caputo model in blue, and the augmented model in black, and the trap compartment ($x_t$, bottom) for Patient 21. The fat compartment concentration ($x_3$) increases progressively, with lower $\alpha _ {31}$ values leading to slower redistribution. The trap compartment ($x_t$), accumulates the drug over time, representing the reservoir effect which leads to delayed release.

The fractional-order parameter $\alpha _{31}$ in the Partial-Caputo model influences the redistribution of Propofol from the fat compartment ($x_3$) into the blood compartment ($x_1$), affecting overall drug kinetics. The term $k_{31}^{\alpha _{31}} {}^{RL}D^{1-\alpha _{31}} x_3(t)$ in the Eq. (21) for $\dot{x}_1(t)$ represents the fractional-order release, where the operator ${}^{RL}D^{1-\alpha _{31}}$ introduces memory effects that slow drug release when $\alpha _{31}$ is small. A lower $\alpha _{31}$ value results in Propofol remaining in the fat compartment for a longer duration, reducing the rate at which it is redistributed into the bloodstream.

Since $x_1(t)$ is directly influenced by the release from $x_3$, a slower release (smaller $\alpha _{31}$) leads to a more gradual increase and sustained levels of Propofol in $x_1$ over time. This slower redistribution affects how quickly Propofol becomes available in the blood for transfer to the effect-site compartment. As a result, $\alpha _{31}$ indirectly affects $x_e$ by affecting the rate at which Propofol is released from adipose tissue into circulation. The drug trapping effect in the augmented model is not apparent in the BIS output during the 6-min simulation, as shown in Fig. 4. However, its influence is visible in the fat compartment, as illustrated in Fig. 6. In the augmented model, the drug concentration is distributed between the fat compartment and the trap compartment. Since the concentration in the trap compartment continues to increase over time, this suggests that its effect may become more pronounced in longer simulations, which will be discussed in the MIMO case. This accumulation could potentially influence drug redistribution and lead to prolonged pharmacodynamic effects.

#### Model testing under clinically relevant dosing protocols

To investigate the robustness of PK models under different anesthetic strategies, we simulated patient 21’s BIS response to three clinically inspired Propofol dosing protocols: (i) a single induction bolus, (ii) repeated bolus, and (iii) continuous infusion. These dosing regimens represent common approaches during surgical anesthesia and were selected to reflect realistic intraoperative practices, where either rapid induction, maintenance through bolus administration, or steady infusion is required depending on clinical goals and patient response.

- *Protocol 1 * In elderly or ASA III–IV patients, induction doses of Propofol are reduced to approximately 1–1.5 mg/kg administered slowly over 60 s to minimize hemodynamic depression and respiratory compromise; for an 88 kg patient, a 100 mg bolus ($\approx$1.14 mg/kg) given over 1 min aligns with published recommendations for older adults, who often require 1–1.5 mg/kg rather than the 2 mg/kg used in younger, healthy individuals^94^.
- *Protocol 2* applies four bolus top-ups of 0.5 mg/kg every 2 min, a typical approach for titrating the effect or responding to transient stimuli. Each bolus (48 mg) was delivered over 20 s, aligning with clinical recommendations for controlled manual injection. Procedural sedation guidelines recommend an initial 0.5–1 mg/kg Propofol dose followed by repeat 0.25–0.5 mg/kg boluses every 2–3 min until the desired sedation level is reached; administering four 0.5 mg/kg ($\approx$44 mg) boluses over 20 s at 2 min intervals conforms to this approach, ensuring gradual titration while monitoring for adverse effects^95^.
- *Protocol 3* uses a continuous infusion at 215 $\mu$g/kg/min, initiated 2 min after the start of simulation and maintained for 18 min. Typical clinical maintenance infusion rates for Propofol range from 100–200 $\mu$g/kg/min (6–12 mg/kg/h) in healthy adults under general anesthesia or monitored anesthesia care (MAC)^94^. In our simulations, an initial rate of 200 $\mu$g/kg/min produced insufficient BIS suppression (BIS remained > 50), so the rate was modestly increased to 215 $\mu$g/kg/min, which is equivalent to approximately 18.9 mg/min for an 88 kg patient, to reliably achieve and sustain a BIS of 40–60 during the procedure.

**Table 6:** Comparison of model performance across dosing protocols on Patient 21. Each row shows the BIS drop time and minimum BIS for a specific model and regimen.

Protocol / Model	$t_{\text {BIS}<50}$ (min)	BIS nadir	Total drug (mg)
One bolus			
Classical	2.1	50.35	100
Augmented	2.1	50.35	100
Fractional ($\alpha =0.5$)	2	49.7	100
Fractional ($\alpha =0.1$)	1.9	48.9	100
Repeated boluses			
Classical	6.63	43.2	192
Augmented	6.63	43.2	192
Fractional ($\alpha =0.5$)	6.56	41.67	192
Fractional ($\alpha =0.1$)	6.53	40.3	192
Infusion			
Classical	20.25	50.6	371.52
Augmented	20.25	50.6	371.52
Fractional ($\alpha =0.5$)	17	47.5	371.52
Fractional ($\alpha =0.1$)	15.9	46.3	371.52

**Fig. 7:**
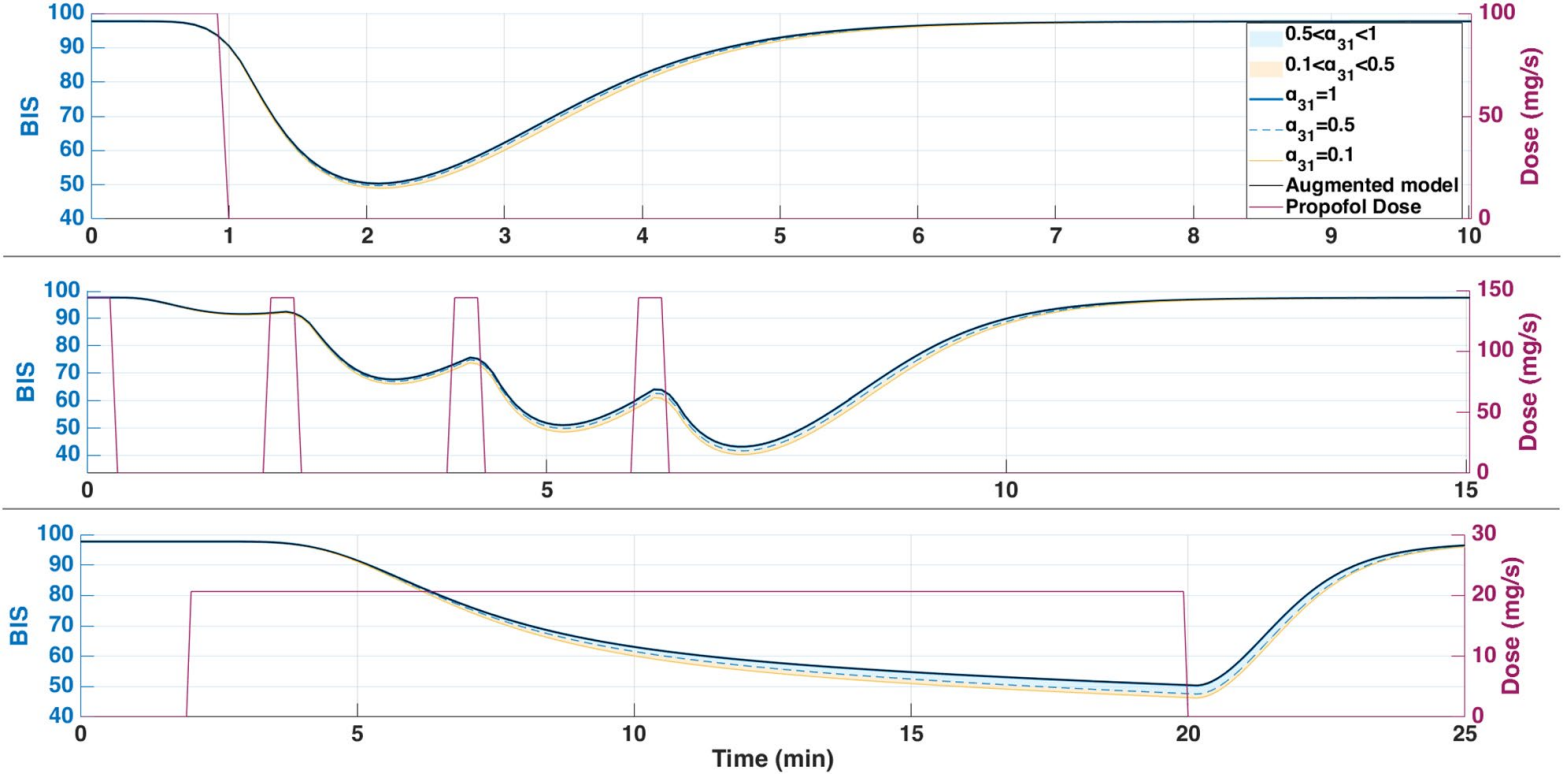
Simulated BIS output under three clinically relevant Propofol dosing protocols for patient 21: (top) single 100 mg bolus over 1 min, (middle) four repeated 0.5 mg/kg boluses every 2 min, and (bottom) continuous infusion at $200\mu \hbox {g}$/kg/min from 2 to 20 min. The magenta curve represents the administered Propofol dose (in mg/s) plotted on the right axis.

**Fig. 8:**
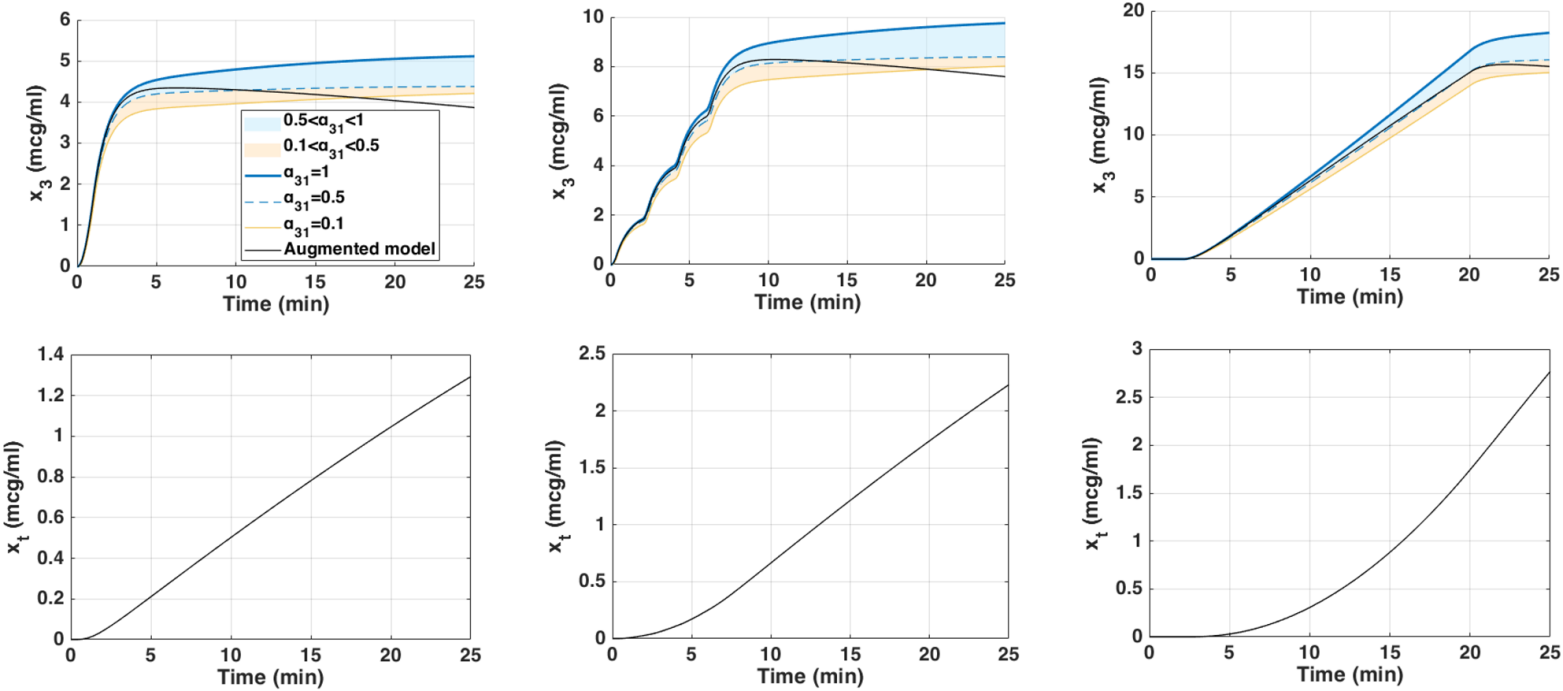
Simulated drug concentration in the fat compartment $x_3$ (top row) and trap compartment $x_t$ (bottom row) across the three dosing protocols: (left) bolus, (middle) repeated boluses, and (right) continuous infusion.

**Fig. 9:**
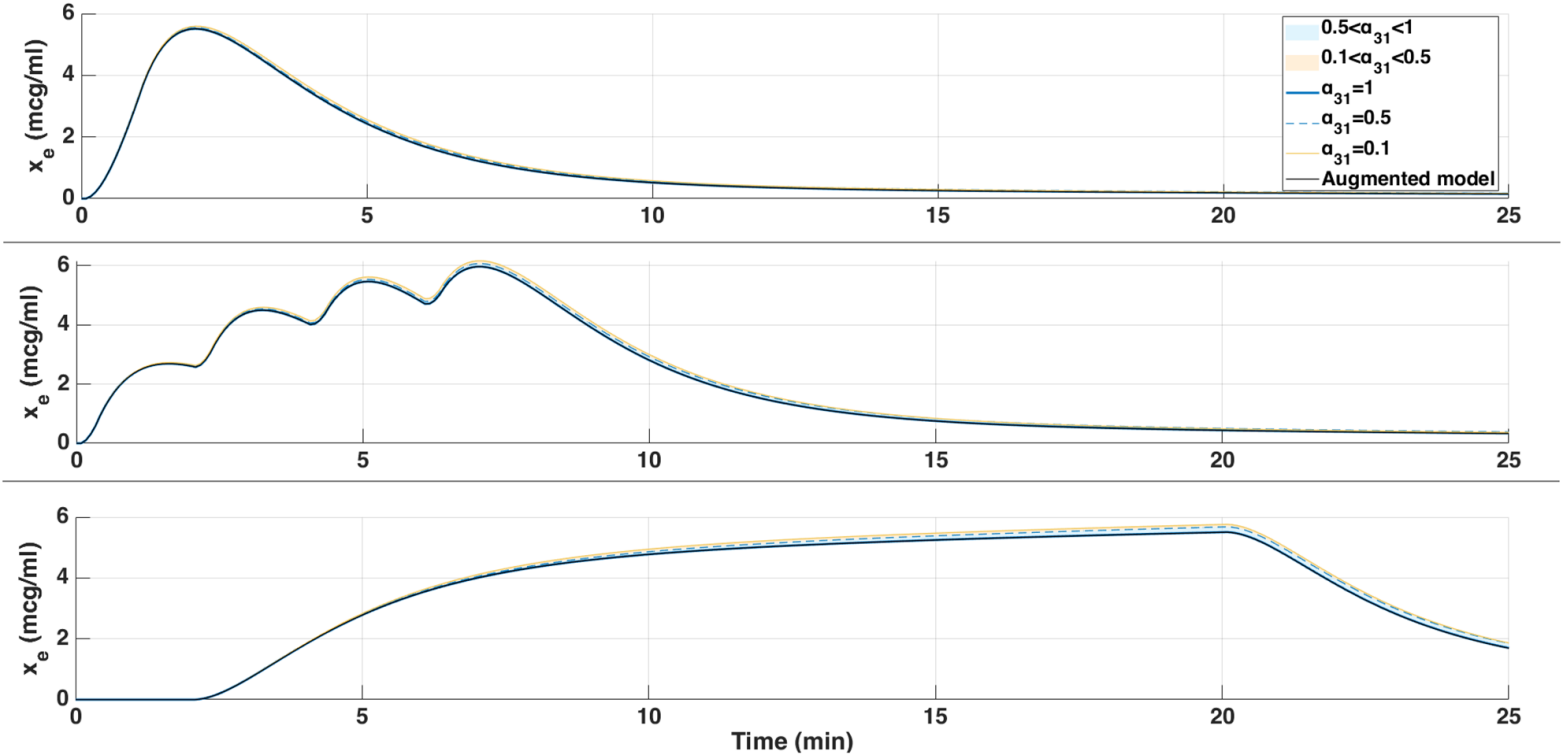
Simulated effect-site concentration $x_e$ for the three dosing protocols: (top) bolus, (middle) repeated boluses, and (bottom) continuous infusion.

Each of these inputs was simulated using three PKc model formulations as previously introduced. The resulting BIS outputs for each dosing protocol are shown in Fig.7, with corresponding fat ($x_3$) and trap ($x_t$) concentrations depicted in Fig. 8, and effect-site concentrations ($x_e$) in Fig. 9. The model performances are shown in Table 6.

From a clinical standpoint, the simulations reinforce well-established practices. A single bolus of 1–1.5mg/kg rapidly induces hypnosis within 2 min but risks overshoot into deep anesthesia, particularly in elderly or hemodynamically unstable patients. Repeated small boluses (0.5 mg/kg every 2 min) offer a slower, more controlled descent into sedation, consistent with titration strategies used in procedural sedation and ASA III–IV cases. Continuous infusion at $215\mu \hbox {g}$/kg/min, though effective for maintenance, proved too slow for induction–requiring up to 20 min to reach adequate BIS suppression. This highlights the clinical need for bolus-induction followed by infusion for maintenance. Interestingly, although the infusion delivers nearly twice the total drug as the repeated bolus protocol (371mg vs. 192mg), it fails to achieve comparable depth, emphasizing that input shape, not just dose, governs clinical response.

From a modeling point of view, key differences emerged. In all protocols, the classical and augmented models gives nearly identical BIS and effect-site trajectories during the first 25 min (Figs. 7, 8 and 9), suggesting that the trap compartment does not influence early dynamics. However, the trap concentration $x_t$ accumulates steadily over time, especially during continuous infusion, revealing its importance in modeling long-term redistribution and potential rebound effects after infusion stops.

Fractional-order models (with $\alpha _{31}=0.1$ or 0.5) differ more noticeably. Lower $\alpha _{31}$ values produce slower onset under infusion protocols, deeper BIS nadirs under bolus protocols, and more prolonged suppression overall. This behavior reflects memory effects, where historical drug presence in peripheral compartments (like adipose tissue) continues to affect central dynamics. Clinically, this mirrors the behavior observed in obese patients, where delayed recovery is attributed to fat sequestration.

These results confirm that fractional models inherently encode tissue retention and delayed release (effects that the augmented compartment tries to mimic explicitly). However, in short protocols, both mechanisms remain latent: their impact is revealed only with sustained inputs or longer simulations.

This three-protocol setup offers a valuable testbed for comparing model robustness. As seen in prior work on cancer therapy dosing^96^, impulse-like inputs (bolus) highlight immediate dynamics, while step-like inputs (infusion) stress the system’s ability to handle accumulation. Our findings echo this: fractional/augmented models show negligible divergence under short impulses but diverge significantly under sustained or repetitive inputs, underlining their importance for long-duration anesthesia modeling. This modeling approach also aligns with previous hypotheses suggesting that certain opioids may undergo tissue-level recycling, thereby sustaining a long-term drug reservoir^10^. Experimental evidence in that study showed that blocking the recycling mechanism significantly accelerated methadone clearance. This highlights its role in prolonging tissue retention and supports the need for models that capture delayed release dynamics.

In summary, these protocoles indicate that short-duration predictions, such as during the induction phase, can be effectively modeled using classical approaches. However, in scenarios involving extended infusions or multi-phase protocols, trap and fractional dynamics become increasingly important. Notably, hybrid protocols combining bolus and infusion may benefit most from incorporating fractional or trap modeling, particularly for patients with high BMI or altered drug clearance.

### MISO case: propofol-remifentanil to BIS

Patient 5428 from the publicly available VitalDB database^97^, which provides high-resolution physiological recordings from surgical patients under general anesthesia, was selected for this case. The biometrics of this patient are presented in Table 7, and the calculated PK parameters are presented in Table 8. The patient’s sensitivity values were estimated using the genetic algorithm described in^22,91^.

The measured BIS data of this patient were also extracted from VitalDB database. BIS signals were recorded using the BIS VISTA monitoring system (Medtronic) with a sampling interval of 1 s Prior to analysis, raw BIS values were preprocessed to exclude artifacts, specifically values equal to 0 or greater than 100, which typically indicate measurement errors or sensor disconnections. Occasional missing data points were linearly interpolated. To enable comparison with PK and PD model predictions, BIS values were resampled and temporally aligned to match the time grid of the simulated effect-site concentrations, using timestamps provided in the VitalDB annotations. This alignment ensured that modeled and measured BIS values could be directly compared over time^97^.

The analysis focuses on the hypnotic/analgesic and the awakening region, as shown in Fig. 3. The surgery region is excluded from this study, as multiple factors such as surgical stimuli, disturbances, complications, or interactions with other drugs, which are not accounted for in the simulation, can influence the BIS index. As previously mentioned in the SISO case, the Partial-Caputo model was solved numerically using a trapezoidal rule-based approach, while the augmented model was simulated with a forward Euler method at a sampling period of $Ts = 1s = \frac{1}{60}$ min. The parameter $\alpha _{31}$ was varied from 0.1 to 1 to study its influence on effect-site concentration and BIS output. The simulation was run for approximately $T=350 min$ corresponding to the duration of the full surgery (full protocol).

**Table 7:** PK model biometric values and PD model sensitivity values of patient 5428.

Index	Gender	Age	Height	Weight	BMI	LBM	$C_{50p}$	$C_{50r}$	$\gamma$	$\alpha$	$E_0$	Surgery
−	−	(years)	(cm)	(kg)	($kg/m^2$)	−	($\mu g/ml$)		−	−	−	−
5428	M	61	166	81	29.4	58.62	4.8053	6	1.6305	0	90.72	$\hbox {AAAR}^{*}$

${*}$ Abdominal Aortic Aneurysm Repair

**Table 8:** Pharmacokinetic parameters for Patient 5428.

$\text {k10}$	$\text {k12}$	$\text {k13}$	$\text {k21}$	$\text {k31}$	$\text {k3t}$	$\text {kt3}$	$\text {ke0}$	$\text {k1e}$	$\text {Cl1}$	$\text {Cl2}$	$\text {Cl3}$	$\text {R}$	$\text {Clt}$	$\text {V1}$	$\text {V2}$	$\text {V3}$	$\text {Vt}$
0.4233	0.2571	0.1958	0.0696	0.0035	0.045	0.0132	1.2	1.2	1.8076	1.098	0.836	0.2654	3.1502	4.27	15.772	238	69.959
0.4716	0.2871	0.0106	0.1667	0.0096	-	-	0.448	0.448	2.329	1.4179	0.0523	-	-	4.27	8.5082	5.42	-

The first row corresponds to Propofol and the second row to Remifentanil.

**Fig. 10:**
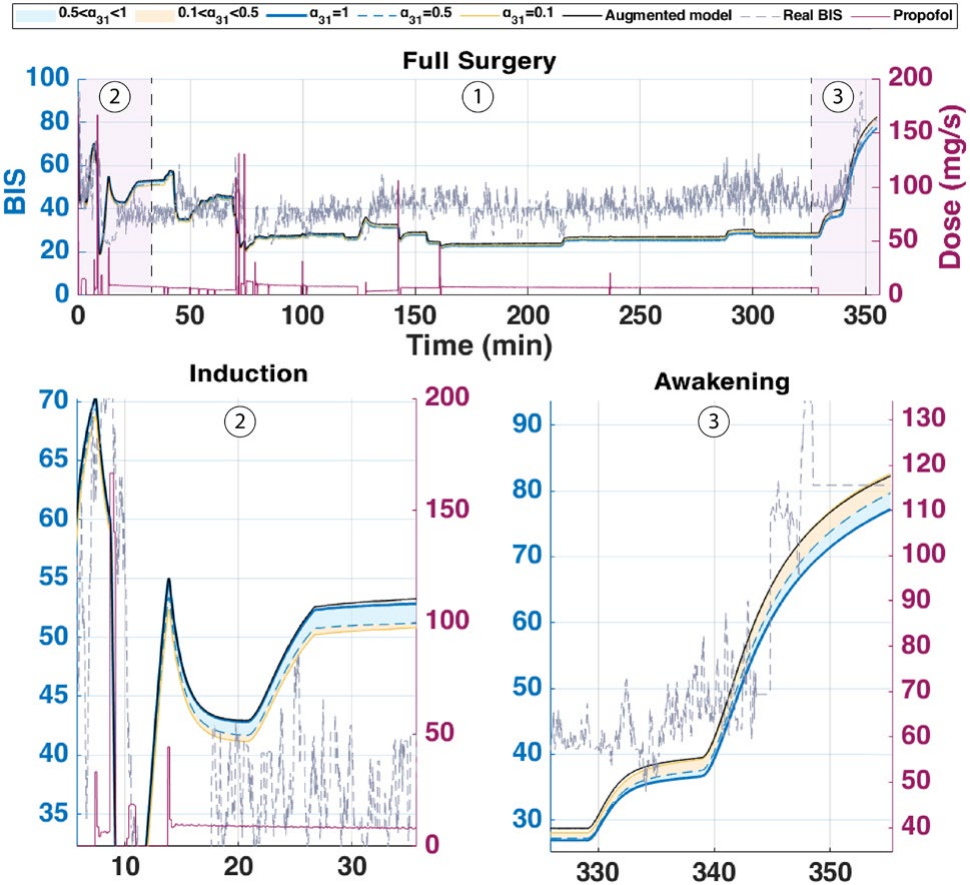
Comparison of measured and simulated BIS responses using different PK models for Patient 5428. The purple curve represents the Propofol input (right axis). The top plot (1) shows the full protocol duration. The bottom left plot (2) zooms into the induction phase (first 10 min, marked with dashed lines) and the beginning of the maintenance phase (up to 30 min, during which Propofol infusion is kept constant), of the hypnotic region. The bottom right plot (3) focuses on the end of surgery and the awakening region, where the Propofol infusion is stopped.

**Fig. 11:**
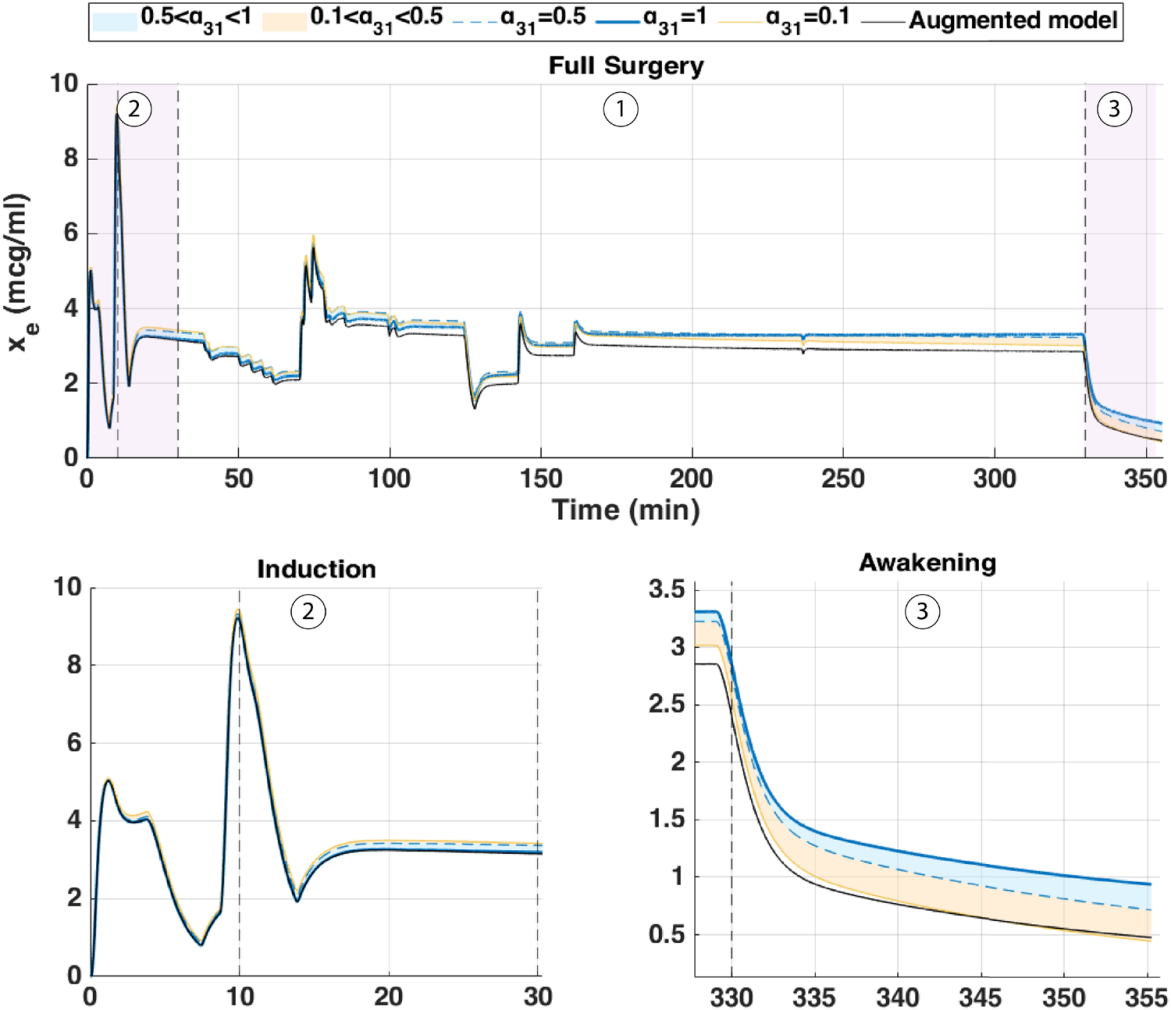
Simulated effect-site concentration ($x_e$ of Propofol) using different PK models for Patient 5428. The top plot (1) shows the entire protocol duration. The bottom left plot (2) zooms into the induction phase (first 10 min, marked with dashed lines) and the beginning of the maintenance phase (up to 30 min), of the hypnotic region. The bottom right plot (3) shows the end of surgery and the awakening phase, where Propofol infusion is stopped.

**Fig. 12:**
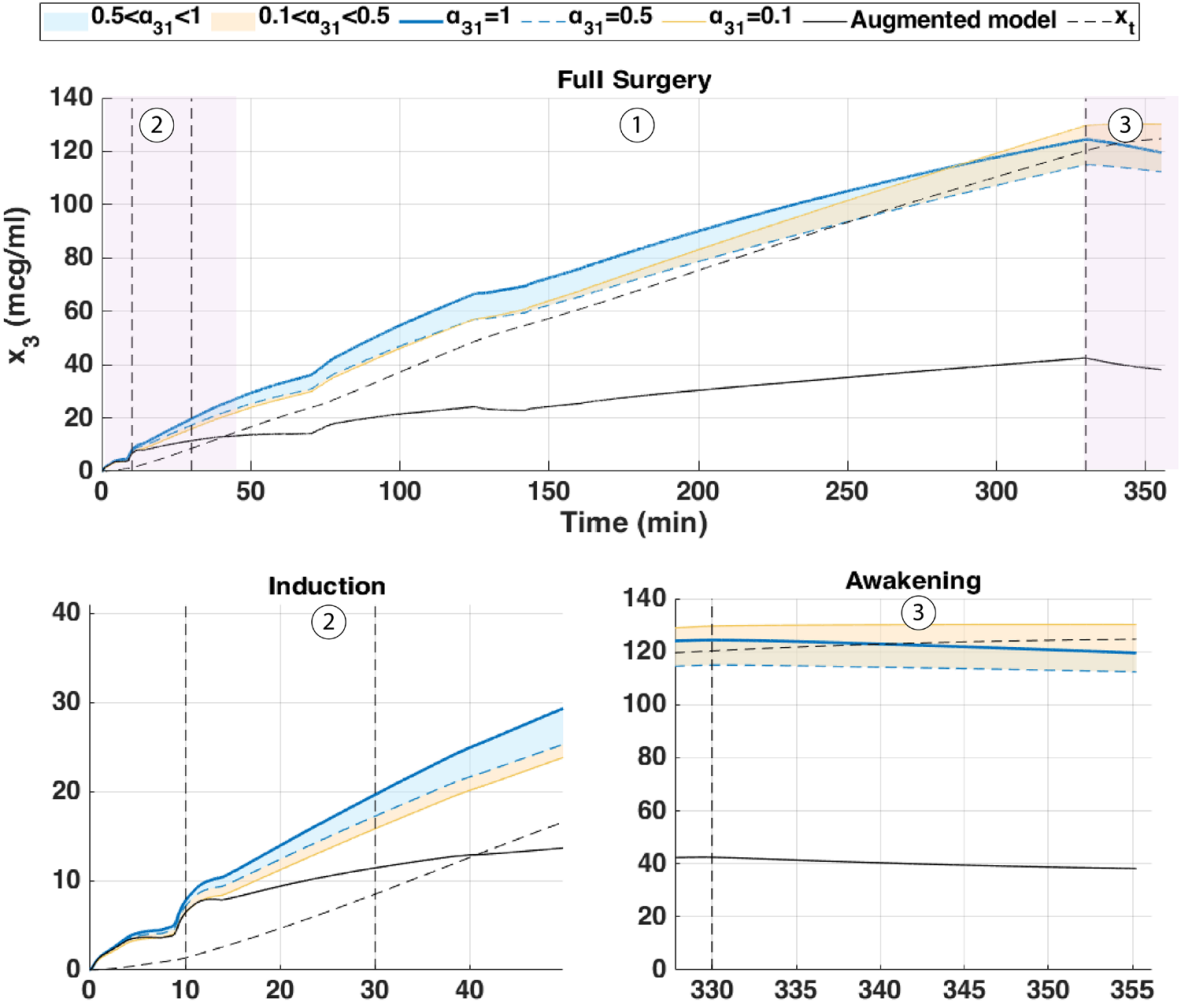
Simulated Propofol concentration in the fat compartment ($x_3$) using different PK models for Patient 5428. The dashed line represents the trap compartment ($x_t$ in the augmented model. The top plot (1) shows the full protocol. The bottom left plot (2) focuses on the induction and early maintenance phases (first 30 min while the bottom right plot (3) shows the end of surgery and awakening phase.

In the hypnotic region, the fractional-order parameter $\alpha _ {31}$ in the Partial-Caputo model affects the redistribution of Propofol from the fat compartment ($x_3$) to the blood compartment ($x_1$), influencing the drug’s overall kinetics. Due to the memory effect introduced by the fractional-order derivative, a smaller $\alpha _ {31}$ leads to a slower release of fat, causing slightly higher concentrations of Propofol in the blood early compared to the classical model, as shown in Fig. 11 (induction). This results in a more pronounced drop in the BIS index and leads to a relatively better match with the measured BIS during induction as shown in Fig. 10 (induction). As the infusion continues, the reservoir effect begins to emerge. Over time, Propofol accumulates in the fat compartment, and its release into the bloodstream slows. Consequently, the plasma and effect-site concentrations gradually decrease, as shown in Fig. 11, which leads to a gradual rise in the BIS signal.

This behavior highlights a dual-phase dynamic: in the short term, a lower $\alpha _ {31}$ slows the absorption of Propofol into fat, resulting in higher concentrations in the blood and a stronger hypnotic effect. However, in the long term, more drug accumulates in the fat compartment, and less drug is available in circulation. This progressive trapping leads to a gradual reduction in the blood and effect-site concentrations of Propofol, causing the BIS level to rise and the hypnotic depth to decrease. The Partial-Caputo model therefore provides a physiologically meaningful way to capture both the initial enhancement and the delayed decline in effect due to redistribution. It performs well during both the induction and awakening phases and begins to diverge from the classical model almost immediately, showcasing the impact of memory-dependent kinetics from the onset of drug administration.

The augmented model introduces an explicit trap compartment to capture the delayed release of Propofol from fat tissue. During the induction phase, the behavior of the augmented model closely resembles that of the classical three-compartment model, and the BIS predictions are nearly identical (Fig. 10). However, as the infusion continues, more drug accumulates in the trap compartment, and its influence becomes increasingly apparent. Around the 40-min mark (Fig. 12), the concentration in the trap compartment exceeds that in the fat compartment ($x_3$), indicating significant accumulation. This causes a noticeable decline in effect-site concentration ($x_e$, Figure 11) and a corresponding rise in BIS (Fig. 10), diverging from the classical model. The augmented model, therefore, begins to better approximate the observed BIS dynamics during and after surgery, especially in longer protocols. This behavior confirms the theoretical reservoir effect described by the model: the trap compartment delays drug return to circulation, gradually reducing the hypnotic effect. By explicitly modeling this delayed release, the augmented model captures longer-term redistribution patterns that become critical in prolonged infusions or recovery periods. This model also showed the slower release of the drug that explains the side effects seen in obese patients in^19^.

#### Performance metrics and error analysis for BIS prediction

To quantitatively assess the predictive performance of the proposed models, we computed standard error metrics including Mean Absolute Error (MAE), Root Mean Square Error (RMSE), and coefficient of determination ($R^2$), as well as correlation coefficients and *p*-values. These metrics were evaluated separately for the induction and awakening phases of anesthesia. The results are shown in Table 9 and 10 for the induction and the awakening phases respectively.

**Table 9:** Performance metrics during the induction phase (0 to 30 min) for Patient 5428.

Model	MAE	RMSE	$R^2$	Corr. coeff.	*P*-Value
Classic / $\alpha _{31}=1$	12.936	16.092	−0.094	0.3671	$1.58 \times 10^{-58}$
$\alpha _{31}=0.75$	12.632	15.849	−0.061	0.3782	$2.73 \times 10^{-62}$
$\alpha _{31}=0.5$	12.157	15.472	−0.011	0.3950	$2.70 \times 10^{-68}$
$\alpha _{31}=0.25$	11.709	15.121	0.034	0.4101	$6.12 \times 10^{-74}$
$\alpha _{31}=0.1$	11.846	15.232	0.020	0.4036	$1.85 \times 10^{-71}$
Augmented	13.026	16.157	−0.102	0.3642	$1.47 \times 10^{-57}$

* P-values refer to Pearson correlation on serial samples and do not imply population-level significance (single patient)

**Table 10:** Performance metrics during the awakening phase (330 min to the end) for Patient 5428.

Model	MAE	RMSE	$R^2$	Corr. coeff.	*P*-Value
Classic / $\alpha _{31}=1$	9.133	10.381	0.657	0.9201	0
$\alpha _{31}=0.75$	8.674	9.956	0.684	0.9201	0
$\alpha _{31}=0.5$	8.002	9.367	0.721	0.9199	0
$\alpha _{31}=0.25$	7.159	8.685	0.760	0.9195	0
$\alpha _{31}=0.1$	6.549	8.164	0.788	0.9191	0
Augmented	6.419	8.037	0.794	0.9192	0

* P-values refer to Pearson correlation on serial samples and do not imply population-level significance (single patient)

**Fig. 13:**
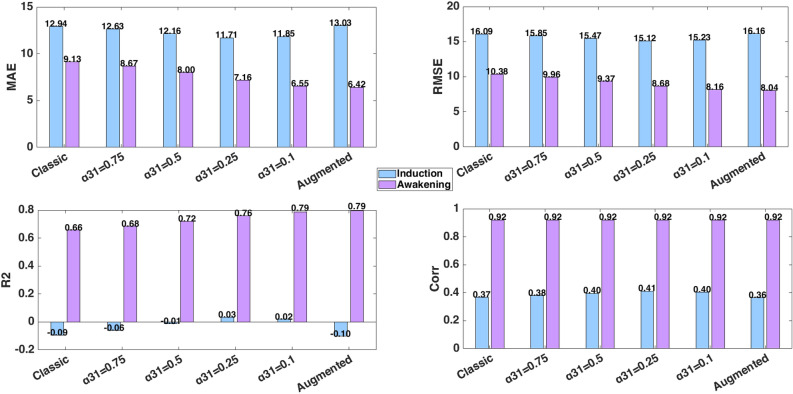
Comparison of prediction performance across models for both induction and awakening phases for Patient 5428. The metrics shown are Mean Absolute Error (MAE), Root Mean Square Error (RMSE), coefficient of determination ($R^2$), and Pearson correlation coefficient (Corr). Each bar represents the model’s performance on either the induction (blue) or awakening (purple) phase. Lower MAE and RMSE values, and higher $R^2$ and Corr values.

To further analyze the error distribution, we generated grouped bar charts for each metric as shown in Fig. 13.

**Fig. 14:**
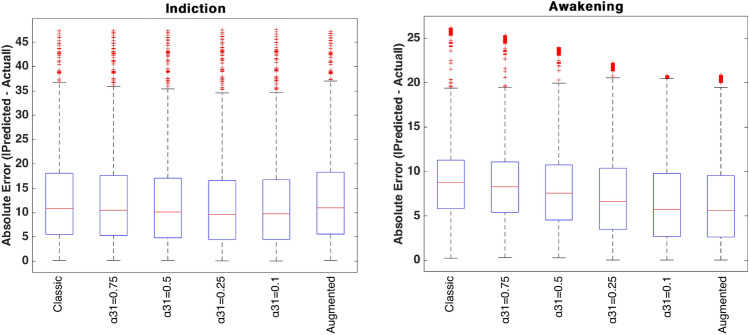
Boxplots of absolute BIS prediction errors for each model during the induction (left) and awakening (right) phases for Patient 5428. Each box represents the distribution of absolute errors ($|\text {Predicted} - \text {Actual}|$) across all time points. Outliers are shown in red.

We also constructed boxplots of absolute prediction errors across all BIS samples as shown in the boxplots in Fig. 14.

Before interpreting the numbers, recall what each metric represents:

- *MAE* is defined as
  $$\textrm{MAE} \;=\; \frac{1}{N}\sum _{t=1}^{N}\bigl |\hat{y}_{t} - y_{t}\bigr |,$$
  where $\hat{y}_{t}$ is the predicted BIS at time *t*, $y_{t}$ is the actual BIS, and *N* is the number of samples. A smaller MAE indicates that, on average, the model’s predictions are closer to the true BIS values.
- *RMSE* is given by
  $$\textrm{RMSE} \;=\; \sqrt{\frac{1}{N}\sum _{t=1}^{N} \bigl (\hat{y}_{t} - y_{t}\bigr )^{2}},$$
  which penalizes large deviations more heavily than MAE. Again, smaller RMSE implies better fit.
- $R^{2}$ measures the fraction of variance in $y_t$ explained by $\hat{y}_t$:
  $$R^{2} \;=\; 1 \;-\; \frac{\sum _{t=1}^{N}(y_{t} - \hat{y}_{t})^{2}}{\sum _{t=1}^{N}(y_{t} - \bar{y})^{2}},$$
  where $\bar{y} = \frac{1}{N}\sum _{t=1}^{N}y_{t}$. An $R^{2}$ close to 1 is ideal; an $R^{2}\approx 0$ means no better than predicting $\bar{y}$; a negative $R^{2}$ indicates the model performs worse than a constant-mean prediction.
- *CorrCoeff* is
  $$r \;=\; \frac{\sum _{t=1}^{N} \bigl (y_{t}-\bar{y}\bigr )\bigl (\hat{y}_{t}-\overline{\hat{y}}\bigr )}{\sqrt{\sum _{t=1}^{N}(y_{t}-\bar{y})^{2}}\;\sqrt{\sum _{t=1}^{N}(\hat{y}_{t}-\overline{\hat{y}})^{2}}},$$
  which ranges in $[-1,1]$. Values near $\pm 1$ indicate strong linear association, while 0 means no linear correlation. The* P* Value tests the null hypothesis of zero linear correlation. A value less than 0.05 indicates that the correlation is statistically significant, suggesting that the observed association is unlikely to be due to random chance.

During the induction phase, the Partial-Caputo model with $\alpha _{31} = 0.25$ demonstrated the best numerical performance among all tested models, achieving the lowest RMSE (15.12) and MAE (11.71), and the highest correlation coefficient (0.41), as shown in Table 9. Although these results suggest an improvement over the classical and augmented models (e.g., RMSE = 16.16 for the latter), the performance gain remains modest. Therefore, while the observed trends are promising, further statistical testing (e.g., paired significance tests or confidence intervals) is warranted to confirm whether these differences are clinically meaningful or arise from numerical variability. At this stage, the findings support an exploratory interpretation, indicating that memory effects introduced via fractional-order modeling may enhance BIS prediction during induction.

In the Awakening Phase, errors decrease for all models. The augmented model shows the smallest MAE (6.42) and RMSE (8.04), with the highest $R^2$ (0.7944), closely followed by the fractional model with $\alpha _{31} = 0.1$ ($R^2 = 0.7878$). These trends are reflected in the box plots (Fig. 14), where both models achieve the lowest median absolute errors and relatively narrow distributions. While all models reach high correlation levels ($r \approx 0.92$), the augmented and fractional ($\alpha _{31}=0.1$) models maintain a slight advantage in overall predictive performance.

The grouped bar charts further support these observations (Fig. 13): the fractional model with $\alpha _{31}=0.25$ ranks best during induction across MAE, RMSE, $R^2$, and correlation metrics; the augmented model performs best during awakening. These comparisons highlight that fractional-order models better handle induction-phase variability, whereas the augmented model aligns more closely with BIS behavior during recovery.

#### Sensitivity analysis

To evaluate the influence of the fractional-order parameter $\alpha _{31}$ on model performance, we conducted a two-stage sensitivity analysis. The goal was to assess how variations in $\alpha _{31}$ affect the root mean square error (RMSE) between the predicted and observed BIS signals, during both the induction and awakening phases of anesthesia.

As an initial step, a local One-At-a-Time (OAT) sensitivity analysis was performed. This classical method involves independently perturbing one parameter at a time while keeping others fixed, and observing the change in model output. For a small increment $\Delta \alpha$, the sensitivity index $S_{\text {OAT}}$ is defined as:

$$S_{\textrm{RMSE}}(\alpha _0) = \frac{|\textrm{RMSE}(\alpha _0 + \Delta \alpha ) - \textrm{RMSE}(\alpha _0 - \Delta \alpha )|}{2\Delta \alpha } \cdot \frac{\alpha _0}{\textrm{RMSE}(\alpha _0)}$$

where $\alpha _0$ is the reference point (or central value) of the parameter $\alpha _{31}$ at which the sensitivity is being evaluated, and $\Delta \alpha =0.1$. This approach is straightforward to implement and provides a direct estimate of the local effect of $\alpha _{31}$. However, it does not account for parameter interactions or variability across the input domain.

To overcome these limitations, we subsequently applied the Morris screening method, a global sensitivity analysis technique that also relies on one-at-a-time perturbations but evaluates effects across multiple randomly sampled points in the input space. The elementary effect (EE) for a given sample point is computed as:

$$\text {EE}_i = \frac{\text {RMSE}(\alpha _{31} + \Delta ) - \text {RMSE}(\alpha _{31})}{\Delta }$$

The Morris method requires specifying a set of configuration parameters, including the number of grid points in the input space, the number of randomly sampled trajectories, and the step size $\Delta$ used to compute the elementary effects. This latter is selected following the standard Morris design:

$$\Delta = \frac{p}{2(p - 1)} = 0.5556 \quad \text {(for } p = 10 \text { grid points)}$$

Table 11 summarizes the values selected for this analysis. The chosen step size of 0.5556 ensures that each perturbation spans approximately 55.56% of the input range, while avoiding boundary overflows due to the symmetric grid. A linear interpolation strategy with extrapolation was employed to estimate RMSE values for perturbed parameter sets.

**Table 11:** Morris method configuration.

Parameter	Value
Grid points (*p*)	10
Trajectories (*N*)	1000
Step size ($\Delta$)	0.5556
Interpolation method	Linear + extrapolate

By repeating this process *N* times for randomly chosen values of $\alpha _{31}$, a distribution of elementary effects is obtained. Two summary metrics are used to characterize the influence of the parameter:

- *The mean of absolute effects*, $\mu ^*$, which estimates the overall (global) influence of the parameter on model error:
  $$\mu ^* = \frac{1}{N} \sum _{i=1}^N |\text {EE}_i|$$
- *The standard deviation*, $\sigma$, which reflects the presence of nonlinear effects or interactions:
  $$\sigma = \sqrt{ \frac{1}{N-1} \sum _{i=1}^N (\text {EE}_i - \bar{\text {EE}})^2 }$$
  where $\bar{\text {EE}} = \frac{1}{N} \sum _{i=1}^N \text {EE}_i$ is the mean (non-absolute) elementary effect.

Therefore, the Morris method provides a more comprehensive picture of parameter importance across the feasible range, making it particularly suitable for models involving fractional-order dynamics.

**Fig. 15:**
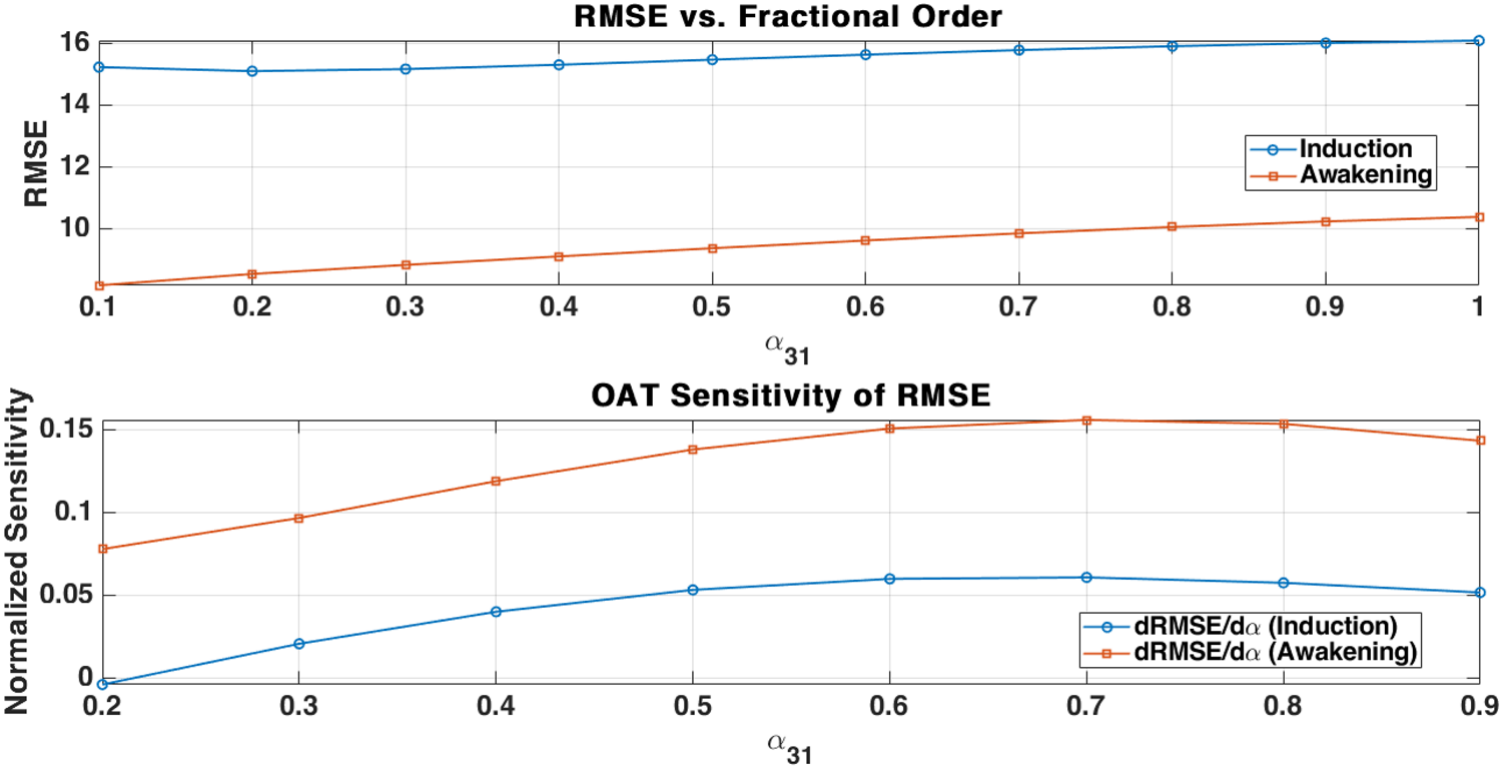
One-at-a-time (OAT) sensitivity analysis of the fractional-order parameter $\alpha _{31}$ with respect to RMSE of Patient 5428.* Top*: RMSE values for induction and awakening phases across a range of $\alpha _{31}$ values.* Bottom*: Normalized OAT sensitivity of RMSE computed using central finite differences. The awakening phase shows a stronger and more consistent sensitivity to $\alpha _{31}$, peaking around $\alpha _{31} = 0.7$, while the induction phase displays a relatively flat and weaker sensitivity profile.

**Fig. 16:**
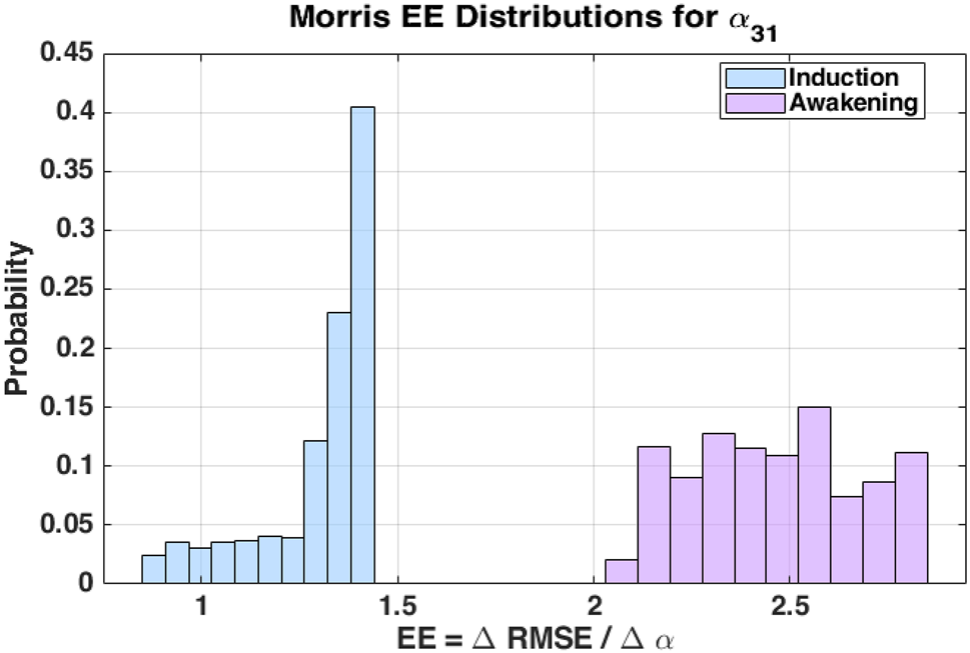
Distribution of elementary effects (EE) for the parameter $\alpha _{31}$ computed via the Morris method, based on RMSE sensitivity of Patient 5428. The awakening phase shows higher magnitude and broader variability of EEs, consistent with greater sensitivity and potential nonlinear effects. Induction-phase EEs are clustered tightly, indicating lower and more consistent impact.

**Fig. 17:**
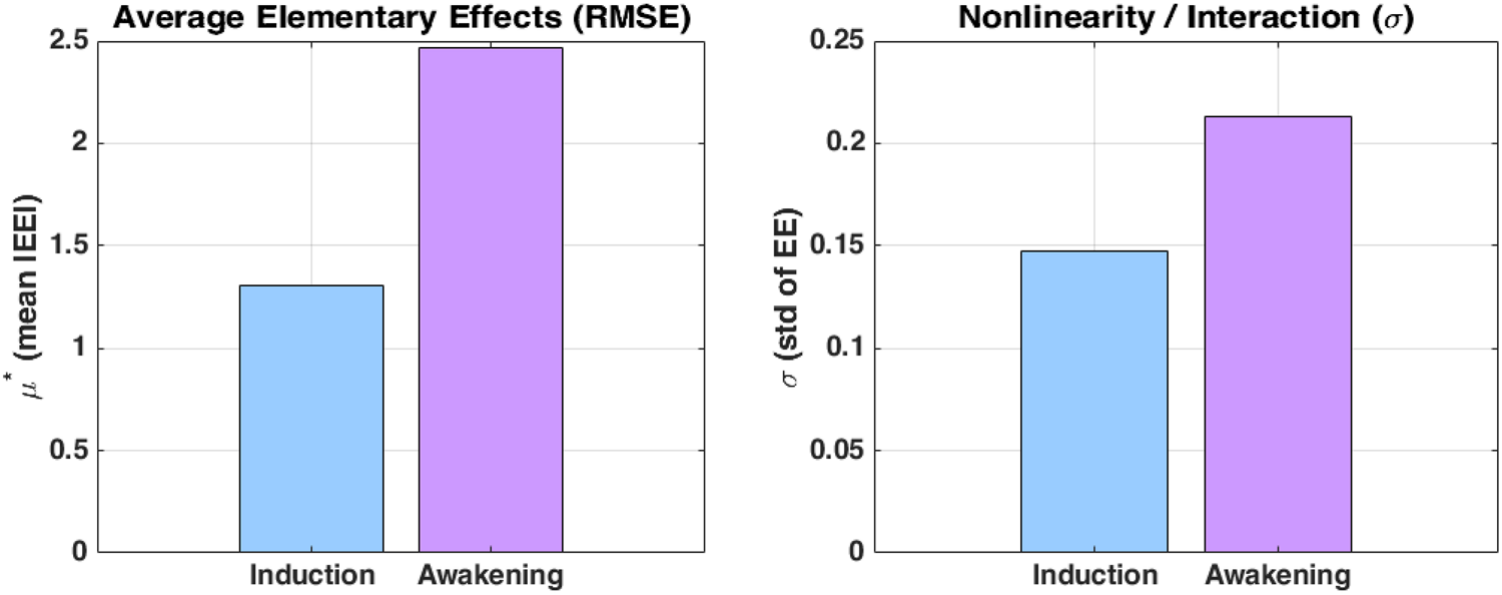
Average elementary effects ($\mu ^*$) and standard deviation ($\sigma$) of the Morris sensitivity analysis for the fractional-order parameter $\alpha _{31}$, evaluated on the RMSE metric (Patient 5428). The results indicate that $\alpha _{31}$ has a stronger and more variable influence on the awakening phase ($\mu ^* = 2.4652$, $\sigma = 0.2131$) than on the induction phase ($\mu ^* = 1.3025$, $\sigma = 0.1474$).

The result of this two analysis are represented by the Table 12 and Fig. 15 for the OAT analysis; and by Figs. 16 and 17 for the Morris Analysis.

**Table 12:** RMSE-based metrics and normalized OAT sensitivities for $\alpha _{31} \in [0.1, 1.0]$ (Patient 5428).

$\alpha _{31}$	Induction metrics			Awakening metrics			OAT sensitivity (Induction)			OAT sensitivity (Awakening)		
	MAE	RMSE	$R^2$	MAE	RMSE	$R^2$	MAE	RMSE	$R^2$	MAE	RMSE	$R^2$
0.1	11.845	15.232	0.0202	6.5487	8.1644	0.7878						
0.2	11.682	15.101	0.0369	6.9783	8.5344	0.7681	−0.0065	−0.0042	0.2210	0.1128	0.0776	−00.0467
0.3	11.769	15.168	0.0284	7.3357	8.8269	0.7520	0.0339	0.0204	−1.4034	0.1438	0.0964	−0.0635
0.4	11.948	15.308	0.0104	7.6816	9.1017	0.7363	0.0649	0.0398	−7.5807	0.1735	0.1187	−0.0850
0.5	12.157	15.472	−0.0110	8.0022	9.3668	0.7207	0.0853	0.0530	9.7306	0.1917	0.1378	−0.1067
0.6	12.362	15.636	−0.0325	8.2953	9.6179	0.7055	0.0952	0.0596	3.7917	0.2003	0.1504	−0.1255
0.7	12.549	15.783	−0.0520	8.5561	9.8491	0.6912	0.0962	0.0605	2.4459	0.1998	0.1557	−0.1389
0.8	12.707	15.909	−0.0688	8.7836	10.056	0.6781	0.0900	0.0572	1.7767	0.1915	0.1533	−0.1453
0.9	12.835	16.011	−0.0826	8.9766	10.234	0.6665	0.0802	0.0514	1.3466	0.1754	0.1431	−0.1429
1.0	12.936	16.092	−0.0936	9.1334	10.381	0.6569						

The results from both OAT and Morris analyses consistently highlight the greater importance of $\alpha _{31}$ during the awakening phase of anesthesia. As shown in Fig. 15, the RMSE increases more steeply with $\alpha _{31}$ in the awakening phase, and the normalized OAT sensitivity peaks near $\alpha _{31} = 0.7$, indicating that fractional-order memory effects play a critical role in capturing post-anesthesia recovery dynamics. In contrast, the induction phase exhibits a relatively flat RMSE curve and low sensitivity, suggesting that $\alpha _{31}$ has limited influence during the onset of anesthesia.

This conclusion is further supported by the Morris analysis. The higher mean elementary effect ($\mu ^* = 2.47$) and standard deviation ($\sigma = 0.21$) for the awakening phase indicate that $\alpha _{31}$ not only has a stronger overall influence but also contributes to variability and possible nonlinearities in model behavior (Figs. 16 and 17). The tight clustering of induction-phase effects, by contrast, reinforces the observation that $\alpha _{31}$ is less critical in modeling this phase.

## Discussion

This section interprets the modeling results in terms of their clinical and methodological relevance. Building on our quantitative findings, we explore the broader implications for anesthesia management, including prediction of awakening, safety in high-risk populations, and integration into control systems and DTs.

### Implications for awakening prediction and safety in anesthesia

The proposed augmented and fractional-order models may enhance DTs with predictive capabilities that can inform anesthesiologists during surgery and in the awakening phase. In particular, these models provide a better representation of long-term drug effects than classical PK/PD models. As illustrated in Fig. 7, the BIS output differences between $\alpha _{31}=1$ and fractional-order cases remain small for single and multiple bolus inputs but become more pronounced during continuous infusion, with the largest discrepancy observed around the 20th min A similar pattern appears in the corresponding effect-site concentrations (Fig. 9), where fractional-order models reveal an increase not captured by classical models potentially leading to misleading predictions if these effects are ignored. Importantly, advanced DTs can leverage these insights not only to help prevent drug overdosing and its associated risks, such as delirium and postoperative nausea and vomiting, but also to enable the development of advanced alarm systems, especially for anti-overdosing alarms, that better represent patient states and improve intraoperative safety. Moreover, these models may also improve prediction of patient recovery: the augmented model achieves the smallest MAE and RMSE during the awakening phase, closely followed by the fractional model with $\alpha _{31}$ = 0.1. Fig. 16 further shows that the awakening phase is substantially more sensitive to $\alpha _{31}$ than the induction phase. This highlights the value of fractional-order dynamics in forecasting awakening from anesthesia and optimizing patient outcomes.

### Application in ICU sedation

Beyond the operating room, the clinical utility of these models extends to prolonged intravenous sedation, such as in the intensive care unit (ICU). Lipophilic drugs are frequently used in ICU sedation protocols and are prone to accumulation in adipose tissue during long-term infusions. This leads to delayed awakening and complicates patient management, particularly during weaning and extubation. The ability of the proposed approaches to capture retention dynamics by explicitly modeling delayed redistribution from adipose tissue align with ICU guidelines. Those guidelines increasingly emphasize light, titratable sedation to reduce delirium and duration of mechanical ventilation. As such, our models may represent a first step toward improving ICU sedation planning, simulation, and control-based optimization strategies.

### Modeling in obese patients and eldery

The ability of our models to capture drug trapping is particularly relevant for high-risk populations. In obese individuals, the expansion of adipose tissue and reduced perfusion amplify drug accumulation, resulting in prolonged pharmacodynamic effects and delayed clearance. The augmented model accounts for this by introducing a trap compartment, while the fractional-order model reflects it through memory-driven dynamics that reproduce the slow release from fat stores. This paves the way for more personalized dosing strategies that improve safety and recovery outcomes in this growing patient population. Although obesity is a primary focus of this study, elderly patients also face heightened risks due to age?related changes in drug metabolism, reduced organ perfusion, and increased sensitivity to anesthetic agents. While our current models do not explicitly include age?specific physiological parameters, they offer a foundation for future investigations. Leveraging our existing dataset, upcoming work should aim to:

1. *Incorporate elderly covariates*: such as reduced hepatic/renal clearance, altered volume of distribution, and heightened PD sensitivity using population PK-PD mixed-effects or PBPK frameworks^98^.
2. *Apply allometric scaling techniques* to adjust dosing and model parameters based on age-related physiological scaling laws^99^.
3. *Validate modified models* using external datasets that include frail or elderly patients^100^ and consider guidelines for “start-low, go-slow” anesthesia strategies.

### Implications for closed-loop control and safety

Efforts to control the depth of hypnosis in anesthesia have advanced significantly in recent years. Although clinical trials are still limited and most systems rely on PID controllers, model predictive controllers (MPC) have demonstrated superior performance in recent studies. They show considerable promise for clinical application, as reported by Pawlowski et al.^101^ and Aubouin-Pairault et al.^102^. However, the effectiveness of MPC depends strongly on the accuracy of the prediction models. In this context, the models presented in this study represent a valuable advancement, with clear potential to enhance MPC-based closed-loop anesthesia systems. The computation of fractional-order models can be demanding at high sampling frequencies. However, the inherently slow dynamics of the patient model and the high noise levels in BIS measurements justify the use of relatively long sampling intervals (typically around 5 to 10 s), which are more than sufficient for the practical application of the proposed models. The use of these models can enable closed-loop studies and clinical trials in major surgical procedures (such as trauma or transplantation), where the maintenance phase is extended. Furthermore, these models can be integrated into existing control strategies to enhance safety mechanisms. This is particularly important for alarm systems, which remain a key concern in the full implementation of closed-loop anesthesia. One promising application is the development of advanced, model-based safety prediction algorithms that can be used to support and improve pre-tuned PID controllers or MPC-based systems.

### Accounting for patients variability

The current model uses BMI as a surrogate for body composition. It adjusts inter-compartmental transfer rates (particularly between the fat and trap compartments) to account for the influence of adiposity on drug retention, as previously discussed in^21^. However, BMI alone does not fully capture the anatomical and functional heterogeneity of adipose tissue. Differences in fat distribution, such as subcutaneous versus visceral adiposity, and vascularization significantly influence drug kinetics^103^. These effects become more pronounced in obese individuals, where increased visceral fat and lower perfusion reduce drug clearance and enhance trapping.

To better represent inter-patient variability, future extensions of the model may incorporate physiological indices such as lean body mass, fat-free mass, or body surface area. Parametrizing the system with such metrics, alongside drug-specific characteristics like lipophilicity (log*P*) (Table 1), could support more physiologically grounded and individualized PK modeling, ultimately enabling model-informed precision dosing in anesthesia.

In terms of clinical relevance, both the fractional-order and augmented models demonstrate the capacity to simulate prolonged drug retention in adipose tissue, as showed previously by the simulated concentrations in the fat and trap compartments during the three protocols (SISO case). In prior 72-h simulations^19,20,22^, the augmented showed delayed decline in drug levels after a bolus injection. This behavior is consistent with the phenomenon of delayed awakening, particularly in patients with high adiposity. While neither model achieves perfect accuracy, their improved performance, especially during the awakening phase as reflected in lower RMSE and higher $R^2$ values.

### Application in digital twins

The adoption of DTs in anesthesia aims to enhance precision medicine by simulating patients responses to anesthetic drugs. They, thereby, supports optimal dosing and minimizing risks such as hypotension, intraoperative awareness, and overdose. However, the implementation of DTs in both anesthesia and broader healthcare applications still faces significant challenges, including limited model complexity, insufficient data for development and validation, and poor scalability, as highlighted by Viceconti et al.^104^. Our study offers a proof of concept that contributes toward addressing some of these challenges by introducing advanced fractional-order and augmented pharmacokinetic models. These models also enable better representation of diverse patient populations (in this case obese patients) which helps expanding the applicability and inclusivity of DTs in anesthetic care.

## Limitation

The augmented model assumes constant inter-compartmental transfer coefficients ($k_{3t}, k_{t3}$), which may not reflect dynamic physiological changes during prolonged anesthesia, such as shifts in perfusion or altered fat metabolism. While the augmented model incorporates a BMI-dependent mechanism for drug trapping, it does not yet account for drug-specific properties like lipophilicity or variable clearance in inflamed or fibrotic fat tissue. Future versions could include lipid solubility–dependent kinetics to better model fat–drug interactions for highly lipophilic anesthetics.

The validation of the model uses BIS to quantify anesthetic depth. However, BIS is a surrogate EEG-based metric influenced by noise, opioid co-administration, and inter-patient variability. These limitations may affect the model’s prediction accuracy, especially during transition phases. BIS also lacks granularity to detect subtle consciousness changes or arousal under neuromuscular blockade.

Validation was performed using data from adult patients under Propofol and Remifentanil infusion protocols. The model does not currently accommodate PK interactions with other agents (e.g. benzodiazepines, inhalational anesthetics), nor does it include compensations for hepatic or renal impairment. These exclusions limit its generalizability to multimodal anesthetic strategies, pediatric settings, or critically ill populations with altered metabolism. In addition, identifying a suitable case for proof-of-concept validation required several constraints: the surgery needed to be sufficiently long to observe and simulate delayed redistribution dynamics; the patient had to be obese; and the data had to include a recorded awakening phase with minimal noise. Such cases are uncommon in publicly available datasets and required extensive manual screening. Because only one patient met all inclusion criteria, the analysis is illustrative for the modeled scenario yet insufficient for statistical generalisation. The findings should therefore be viewed as hypothesis-generating until validated in larger, population-level cohorts.

Although the ODE-based augmented model improves numerical stability and enables discrete-time simulation, the inclusion of a trap compartment introduces additional parameters that must be estimated from data. This increases the identification complexity and can hinder real-time applicability unless robust estimation techniques are employed. Similarly, while fractional-order models better capture memory effects, they are computationally intensive and not yet optimized for embedded control environments.

While the current study demonstrates the physiological plausibility and modeling advantages of fractional and augmented pharmacokinetic models, the evaluation was performed on a limited number of representative patient cases. These cases were intentionally selected to explore and illustrate model behavior under specific anesthetic protocols, and the study should be regarded as a proof-of-concept rather than a population-level validation. As such, statistical measures of inter-individual variability, such as standard deviations or confidence intervals across a broader cohort, were not included. This constraint limits the ability to generalize the findings or to draw definitive conclusions about model superiority over classical three-compartment formulations. Future studies will address this limitation by including larger and more diverse patient cohorts, enabling statistical validation of model performance and enhancing generalizability across different clinical scenarios.

## Future work

The present study serves as a proof of concept using a small number of carefully selected high-resolution clinical cases. Although these were sufficient to demonstrate the physiological plausibility and modeling advantages of augmented and fractional-order pharmacokinetics, they are insufficient for broad validation or generalization. As a next step, we plan to build a large-scale dataset that includes diverse patient profiles by age, BMI, ethnicity, and comorbidity. This data set will allow robust statistical validation, the evaluation of population heterogeneity, and the reduction of center-specific biases. Furthermore, harmonized extraction of drug infusion profiles, EEG, and hemodynamic waveforms will support external validation of the proposed models. Such efforts are essential to establish clinical applicability and improve model robustness.

One important factor to consider is the interaction between the drug and the patient’s cardiovascular state. Propofol infusion leads to a decrease in cardiac output, which reduces hepatic perfusion and slows the drug’s clearance^105,106^. This effect is not captured by classical PK models that assume fixed clearance rates. During the induction phase, for example, a reduction in clearance increases the effect-site concentration, which in turn lowers the predicted BIS value. If this behavior is incorporated into the model, the simulated BIS output presented in the previous section could align more closely with the deeper sedation levels observed in clinical data during induction. Introducing a dynamic scaling of clearance based on cardiac output may therefore improve the model’s ability to capture the onset of anesthesia more accurately. Additionally, the rapid onset of Propofol’s hypnotic effects cannot be fully explained by first-order effect-site models. Clinical observations show that BIS and EEG responses often occur faster than conventional effect-site compartments predict. The discrepancy between clinical observations and conventional model predictions suggests that the effect-site dynamics could be more accurately captured using a fractional-order model, or that classical models underestimate its rate of uptake and redistribution^107^. Incorporating fractional-order kinetics offers a physiologically plausible and mathematically compact way to capture these fast and history-dependent transitions.

Future work could also extend the current intravenous-only PK/PD framework to encompass multi-drug interactions, particularly combining inhaled agents with IV anesthetics. Inhaled agents require additional compartments in the PK model to reflect pulmonary uptake, determined by alveolar concentration and blood–gas partition coefficients, which are influenced by cardiac output and tissue solubility. For example, Propofol-induced hypotension may alter Sevoflurane clearance, creating interdependent PK feedback loops that are not captured in static compartment models^108^. Moreover, due to their high lipid solubility, inhaled agents like Sevoflurane equilibrate slowly in adipose tissue, potentially prolonging awakening in obese patients. Incorporating these dynamics would enhance the model’s ability to simulate anesthetic protocols and improve predictions of recovery and interaction effects.

## Perspective

General anesthesia induces complex, nonlinear physiological changes in the human body, particularly as patients transition into deeper states of unconsciousness. These changes challenge the assumptions of classical PK models, which often rely on fixed compartmental structures and constant clearance rates. While such models approximate patient responses in the awake state, they struggle to capture the dynamic behaviors that emerge during DoH^109^, especially in patients with comorbidities^110^ or those with altered hemodynamics.

The findings of this paper point toward the need for more physiology-aware models, capable of representing both short-term diffusion and long-term retention effects, especially in adipose tissue. The Euler-based analysis shows that adding a compartment induces fading memory behavior in the fat compartment, structurally similar to fractional-order systems. This suggests that biological memory effects, such as delayed drug release from fat, can be modeled without fractional calculus. This effect is particularly visible during the maintenance phase, where the BIS predicted by the augmented model is slightly higher than that of the classical model, bringing it closer to clinical recordings. However, the model still underestimates BIS during this phase, suggesting that additional intraoperative factors may be influencing consciousness levels, which should be further investigated and modeled.

In this context, DTs emerge as a natural extension of such models. DTs can act as rich data sources for machine learning, optimization, and closed-loop control systems^90,111,112^. Previous work by Pawlowski et al.^113^ demonstrated the feasibility of such clinical integration through a DT interface successfully applied in closed-loop anesthesia trials. Beyond prediction and control, DTs have potential as simulation environments for training and clinical rehearsal^114^. Anesthesiologists could use twins to explore how different patients (comorbidities, elderly, or hemodynamically unstable) respond to various drug protocols. These in silico simulations may help identify safe operating windows, reduce awakening delays, and personalize sedation strategies in complex cases. Another aspect could be the simulation of therapeutic interventions without the ethical and logistical constraints of traditional trials. This approach holds the potential to substantially reduce costs by minimizing patient recruitment burdens, streamlining protocol design, and decreasing the number of in vivo measurements required. Moreover, DTs mitigate infrastructural constraints by enabling remote hypothesis testing and iterative experimentation in silico. In the long term, they may facilitate adaptive trial designs, accelerate regulatory evaluations, and enable personalized treatment optimization.

Building on this concept, our team is currently developing next-generation user interfaces to support the clinical application of DTs and closed-loop control. These interfaces will incorporate sophisticated patient model selection tools, enabling representation of the broader patient population addressed in this study. Furthermore, we are integrating advanced and precise alarm and safety systems into both existing and new control and digital twin platforms. The resulting interfaces will serve multiple purposes: supporting clinical use, providing educational tools for anesthesiologists, and facilitating research studies.

## Conclusion

This study addressed the limitations of classical PK models in capturing delayed drug redistribution in adipose tissue, particularly in the context of general anesthesia with Propofol. To better represent the history-dependent dynamics observed in fat compartments, we proposed two physiologically grounded extensions: an augmented model incorporating a trap compartment, and a fractional-order model using Caputo derivatives.

Euler discretization revealed that the augmented model exhibits fading-memory behavior, akin to the fractional-order model, suggesting their shared ability to model long-term retention. Both models were integrated into a PK–PD framework and evaluated against BIS measurements in clinical TIVA protocols for obese patients. Both models showed lower RMSE than the classical framework during BIS prediction, particularly under sustained dosing conditions. The augmented model captured delayed redistribution more accurately during the awakening phase, while the fractional-order model improved early-phase predictions. These findings highlight the physiological relevance of memory effects in anesthesia modeling and suggest a path forward for the development of DTs. By bridging pharmacological modeling with nonlinear dynamical systems, this work could inform future efforts toward individualized anesthesia management, pending broader validation.

Quantitative evaluation showed that both memory-aware models improved BIS prediction accuracy relative to the classical three-compartment model. During the awakening phase, the augmented model lowered RMSE from 10.38 to 8.04 (−22.5%) and increased $R^2$ from 0.66 to 0.794, while the best fractional-order configuration reduced RMSE to 8.16 (−21.4%) with $R^2$ of 0.788. Moreover, sensitivity analysis revealed that BIS dynamics during awakening were substantially more influenced by $\alpha _{31}$ than during induction, underscoring the importance of modeling memory effects.

While the proposed models improve BIS prediction by incorporating memory effects in adipose tissue, several simplifications remain. In particular, intraoperative physiological changes such as nociceptive stimulation, autonomic responses, and dynamic clearance rates were not included. Such intraoperative dynamics may partially explain the residual mismatch between simulated and clinical BIS during the maintenance phase. Future extensions could address these dynamics by integrating cardiovascular feedback, analgesic modeling, and surgical stimulation.

It is important to note that this modeling approach is intended as an initial step toward gaining physiological insight. The main goal of this study is to explore how delayed drug release from adipose tissue might be represented. To do this, we used fractional derivatives to reflect the long-memory effects seen in drug redistribution. We then compared the behavior of this fractional model with our augmented model. This comparison helps us explore whether the observed drug retention could be explained by a specific anatomical structure or if it is better captured through a more general memory-based approach. Although the results are preliminary, the similarity between the two models suggests that the trap model may be a reasonable starting point for further studies and controller development.

## Figures and Tables

**Algorithm 1:**
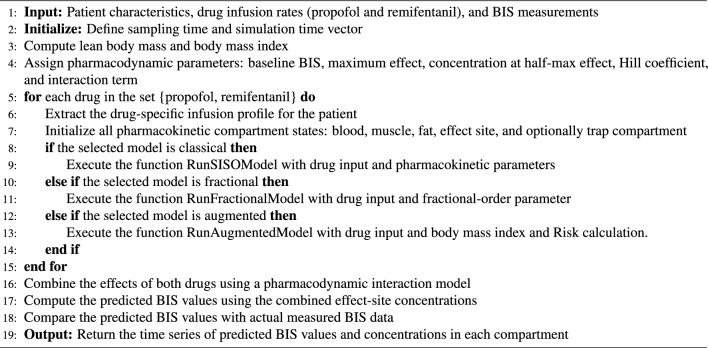
MISO PK–PD Simulation Framework

**Algorithm 2:**
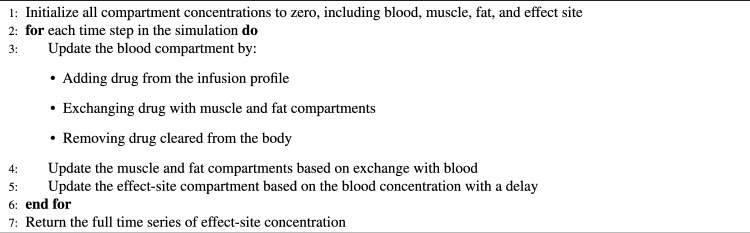
RunSISOModel: Single Drug Simulation with Classical Pharmacokinetic Model

**Algorithm 3:**
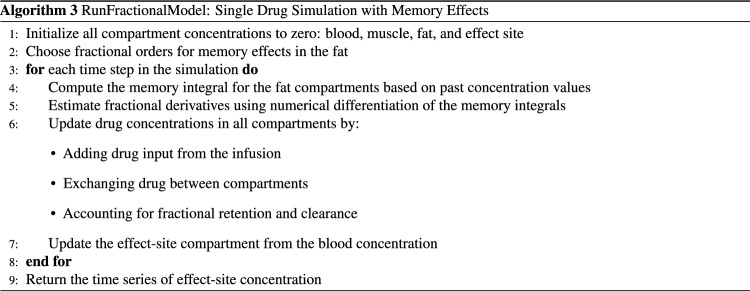
RunFractionalModel: Single Drug Simulation with Memory Effects

**Algorithm 4:**
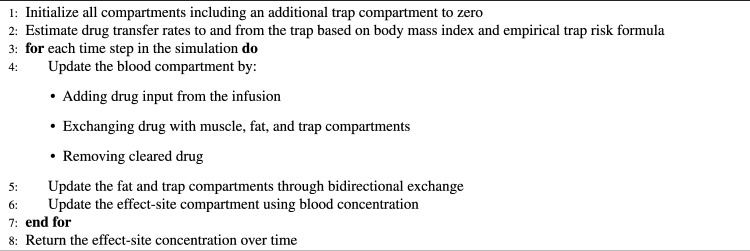
RunAugmentedModel: Single Drug Simulation with Trap Compartment

## Data Availability

The dataset for patient 5428 used in this study is publicly available from the VitalDB database at https://vitaldb.net/dataset. Simulation results for both patients 21 and 5428 were generated using the patient simulator described in^90^, available on Zenodo at https://zenodo.org/records/14014536. Biometric data for patient 21 were published in^91,115^. Additional information is available from the corresponding author upon reasonable request.

## A Binomial expansion

The binomial expansion formula states that:

28 $$\begin{aligned} (a + b)^n = \sum _{j=0}^n \left( {\begin{array}{c}n\\ j\end{array}}\right) a^{n-j} b^j. \end{aligned}$$

Setting $a = 1$ and $b = -k_{t3}h$, the expression becomes:

29 $$\begin{aligned} (1 -k_{t3}h)^{i-1} = \sum _{j=0}^{i-1} \left( {\begin{array}{c}i-1\\ j\end{array}}\right) (-1)^j (k_{t3}h)^j. \end{aligned}$$

Introducing the change of index $j = k - 1$, the sum in (29) can be rewritten as:

30 $$\begin{aligned} (1 -k_{t3}h)^{i-1} = \sum _{k=1}^{i} \left( {\begin{array}{c}i-1\\ k-1\end{array}}\right) (-1)^{k-1} (k_{t3}h)^{k-1}. \end{aligned}$$

Multiplying both sides of (30) by $k_{t3}h$ gives:

31 $$\begin{aligned} k_{t3}h(1 -k_{t3}h)^{i-1} = \sum _{k=1}^{i} \left( {\begin{array}{c}i-1\\ k-1\end{array}}\right) (-1)^{k-1} (k_{t3}h)^{k}. \end{aligned}$$

## Notation Summary

The Table 13 shows a summary of all sympbols, their discription and their corresponding units.

**Table 13:** Summary of model symbols and corresponding physical units.

Symbol	Description	Unit
*u*(*t*)	Drug infusion input	[mass]/[time]
$x_1, x_2, x_3$	Drug concentration in blood, muscle, and fat compartments	[mass]/[volume]
$x_t$	Drug concentration in trap compartment	[mass]/[volume]
$k_{ij}$	Transfer rate from compartment *i* to *j*, $i \ne j$	1/[time]
$k_{3t}, k_{t3}$	Transfer rates between fat and trap compartments	1/[time]
$k_{1e}, k_{e0}$	Plasma to effect-site and effect-site equilibration rates	1/[time]
$V_1, V_2, V_3$	Volume of blood, muscle, and fat compartments	[volume]
$C_{l1}, C_{l2}, C_{l3}$	Clearance rates of compartments 1–3	[volume]/[time]
$\alpha _{ij}$	fractional-order for drug transfer between compartments *i* and *j*	–
$D^\alpha$	Caputo or Riemann–Liouville fractional derivative of order $\alpha$	–
$I^{\alpha }$	Riemann–Liouville fractional integral of order $\alpha$	–
$E_0$	Baseline effect	Depends on effect index
$E_{\max }$	Maximum achievable drug effect	Depends on effect index
$C_{50}$	Concentration at half-maximal effect	[mass]/[volume]
$\gamma _p$	Hill coefficient describing curve steepness	–
*BMI*	Body mass index ($W/H^2$)	kg/$\hbox {m}^2$
*BIS*	Bispectral Index (clinical measure of hypnosis)	–
*R*	Risk indicator for drug trapping in adipose tissue	–
